# Small-scale fisheries in ecologically sensitive areas in Latin America and the Caribbean: Do marine protected areas benefit fisheries governance?

**DOI:** 10.1007/s13280-024-02062-z

**Published:** 2024-09-06

**Authors:** Ana Cinti, Luisa Ramirez, Mauricio Castrejón, Jaime A. Aburto, Luciana Loto, Stuart Fulton, Mario Rueda, Alexandre Schiavetti, Francisco J. Fernández-Rivera Melo, Manuel Bravo, Daniela Trigueirinho Alarcon, Valéria Penchel Araújo, Ana M. Parma

**Affiliations:** 1 Center for the Study of Marine Systems (CESIMAR), CCT CONICET-CENPAT, Blvd. Brown 2915, CP 9120 Puerto Madryn, Chubut Argentina; 2 Fisheries and Oceans Canada, 900 Tolmie Avenue, Victoria, BC Canada; 3 Grupo de Investigación en Biodiversidad, Medio Ambiente y Salud, Universidad de Las Américas, UDLAPark, redondel del ciclista s/n, Quito, Ecuador; 4 Departamento de Biología Marina, Facultad de Ciencias del Mar, Universidad Católica del Norte, Larrondo 1281, Coquimbo, Chile; 5 Department of Environmental Management, National University of Moreno, Av. Bartolomé Mitre 1891, B1744OHC Moreno, Provincia de Buenos Aires Argentina; 6 Comunidad y Biodiversidad (COBI) A.C., Isla del Peruano 215, Lomas de Miramar, CP 85448 Guaymas, Sonora Mexico; 7 Institute of Marine and Coastal Research (INVEMAR), Calle 25 No. 2-55, Playa Salguero, Santa Marta D.T.C.H., Magdalena, Colombia; 8 LECAP - DCAA - UESC, Universidade Estadual de Santa Cruz, Rod Jorge Amado km 16, Salobrinho, Ilheus Bahia 45662-900 Brazil; 9 WILDAID, Colinas de Los Ceibos, Av. Octava 302 y Calle Séptima, Guayaquil, Ecuador; 10 Independent Consultant in Socio-Environmental Projects, Rua Francisca Lopes de Souza, 148, casa 04, Itaipu, Niterói, Rio de Janeiro CEP 24344-175 Brazil

**Keywords:** Governance approach of marine protected areas, Institutional design, Latin America and the Caribbean, Marine protected areas, Small-scale fisheries governance

## Abstract

Many small-scale fisheries (SSFs) in Latin America and the Caribbean (LAC) operate in ecologically sensitive areas, where balancing conservation and resource use is challenging. 'Multiple-use' marine protected areas (MPAs) have been implemented to accommodate SSFs (among other uses) within areas designated for conservation, creating opportunities and challenges for SSF governance. We analyzed eleven case studies from LAC to explore: (1) how different MPA institutional designs affect key aspects of SSF governance and (2) the links between these effects and the type of initiative that promoted MPA establishment (origin). Results indicate that the existence of an MPA benefited SSF governance in many ways, with more pronounced positive effects in MPAs with mixed to bottom-up origin supported by well-organized fishing groups. In addition, the presence of supportive MPA authorities that leveraged local capacities and initiatives and adopted flexible and collaborative governance systems made a difference in several cases. Lessons for integrating MPA and SSF governance are drawn.

**Supplementary Information:**

The online version contains supplementary material available at 10.1007/s13280-024-02062-z.

## Introduction

Small-scale fisheries (SSFs) provide food and livelihoods to millions of people worldwide, with at least 60 million people employed —formally and informally—along the value chain, accounting for 40% of the global catch and 90% of all employment in capture fisheries worldwide (FAO 2021). Marine SSFs occur in coastal areas and are likely to be affected by and compete with diverse uses of the coastal zone (e.g., urbanization, tourism, biodiversity protection). Different societal sectors perceive coastal ecosystems to be valuable in different ways, which often leads to conflicts (Jones 2002).

Many SSFs in Latin America and the Caribbean (LAC) operate in ecologically sensitive areas, which are formally protected as multiple-use marine protected areas (MUMPAs) (Guarderas et al. 2008), a category of marine protected area^1^ (MPA), equivalent to IUCN category VI, designed to accommodate diverse objectives (e.g., conservation, resource use, recreation) using various management tools (e.g., no-take zones, seasonal closures, catch/gear restrictions) under unifying institutional frameworks (Agardy et al. 2003; The World Bank 2006). The designation as MUMPAs admits an enormous diversity of institutional designs worldwide and follows a global trend toward the implementation of more inclusive protected areas (PAs) for human rights/welfare reasons, but also for functional reasons (e.g., benefits of participation) (Naughton-Treves et al. 2005; Day et al. 2012). In addition to MUMPAs, areas under different institutional regimes subject to the so-called Other Effective area-based Conservation Measures (OECMs) often combine various goals (conservation, fisheries enhancement, reinforce traditions) and many are led by communities through partnerships with governments and civil society organizations (Jupiter et al. 2014; Dudley 2018).

Diverse formats of MUMPAs have been implemented in LAC to accommodate SSFs—among other uses—within areas designated for conservation. Some examples comprise Extractive Reserves (ER) in Brazil, Regional Integrated Management Districts (RIMD) in Colombia and Biosphere Reserves in Mexico (Orensanz et al. 2013). The diversity of institutional arrangements is paralleled by a diversity of goals and objectives and of governance^2^ approaches, which range from top-down (government led) to bottom-up (community led) systems. MPAs established through bottom-up processes have been mainly created to secure the rights of indigenous/local communities to maintain their livelihoods and traditions, and to exclude large-scale economic activities such as industrial fishing, aquaculture farms and large 'development' projects (e.g., real estate, industries, ports and roads construction). By contrast, environmental protection has been the primary motivation for the top-down designation of MPAs, with more stringent restrictions on extractive activities. External drivers have also influenced the top-down creation of MPAs, which tripled in global extension following the declaration of Aichi Target 11 (CBD 2018; UNDP et al. 2021) and are likely to be expanded further under the 30 by 30 agenda.^3^ International conservation targets have prioritized area coverage over effective governance and equity (Thomas et al. 2014; MacKinnon et al. 2021), encouraging rapid top-down designations without adequate planning and consultation (see Gaymer et al. 2014; Kriegl et al. 2021). Protected areas are unlikely to achieve the twin goals of biodiversity conservation and poverty alleviation without effective management (Fox et al. 2014). Widespread shortfalls in human and financial resources have been identified as major constraints to effective and equitable management in many MPAs (Gills et al. 2017).

Both, top-down and bottom-up MPA governance approaches have strengths and weaknesses (Jones and Long 2021), and there is now widespread recognition that they should be combined through collaborative forms of governance (Kelleher 1999; Jones 2002; McCay and Jones 2011; Gaymer et al. 2014; Jones and Long 2021). Top-down governance offers the power and resources of the state and the potential for governance across larger areas, while bottom-up governance empowers members of civil society by involving them directly, either as autonomous decision-makers or as partners with government (McCay and Jones 2011).

In ecologically sensitive settings, conservation initiatives have created opportunities but also challenges for SSFs governance. MPAs may affect fisheries in various aspects, including rules of access and resource use, environmental stewardship, enforcement and monitoring, decision-making processes, fundamental rights recognition and socioeconomic impacts. In this paper, we review various experiences with multiple-use MPAs from LAC, all of which have SSFs operating inside their boundaries, to investigate the effects of diverse formats of MPAs on relevant aspects of SSFs governance (e.g., access, tenure security, stewardship, decision making, enforcement). We hypothesize that the type of process through which the MPA was established has a role in determining whether the MPA has a beneficial or disruptive effect on fisheries governance. Although the emphasis is on fisheries, we provide insights for improving the effectiveness of the overall MPA governance. The aim is to identify strategies that synergize the conservation and sustainable use of marine ecosystems and resources in MPA settings.

## Methods

**Fig. 1:**
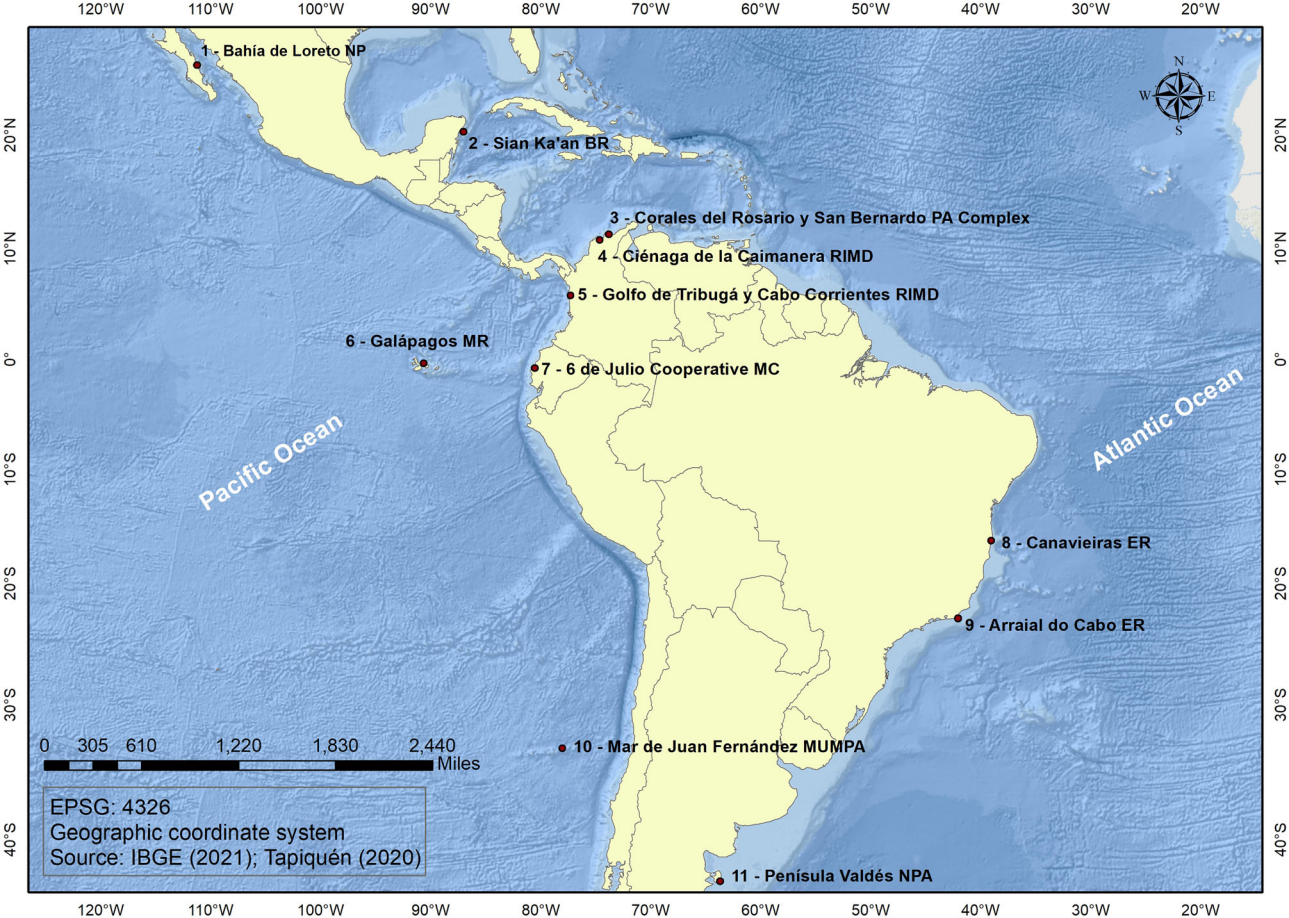
Study area and case studies. BR: Biosphere Reserve; ER: Extractive Reserve; RIMD: Regional Integrated Management District; MC: Mangrove Custodia; MR: Marine Reserve; MUMPA: Multiple-Use Marine Protected Area; NPA: Natural Protected Area; NP: National Park

All case studies selected represent MPAs aimed at conservation among other goals, and that function in practice as multiple-use MPAs, regardless of their formal designation category. They comprise 11 cases from Mexico, Colombia, Ecuador, Brazil, Chile, and Argentina, with which we have first-hand experience. They present a range of governance approaches (top-down, mixed, bottom-up, as defined below), institutional designs (e.g., arrangements for decision-making) and fisheries management systems (e.g., use-rights granted to fishing groups or communities, individual licenses, traditional tenure systems) (see Fig. 1 and Table 1). The MPAs vary greatly in extension, from about 20 to 13,000 km^2^, and most cover land and marine or estuarine territory. In most cases local fisheries target benthic resources (shellfishes, sea cucumbers, lobsters, crabs); only a few have fish species as main targets (e.g., Arraial do Cabo ER, Ciénaga de la Caimanera RIMD). Additional information is summarized in Table 1 and Table S1 (supplementary material). All but one case study, the Mangrove *Custodia*, are recognized in national or provincial legislation as protected areas. However, *Custodias* had an explicit conservation goal and could qualify as OECM. Results are case-specific and should not be interpreted as representative of the region as a whole or of individual countries included in the analysis.

**Table 1:** Basic attributes of case studies, arranged from North to South

Country / MPA and year created	Trigger for MPA creation / MPA origin	MPA objectives	MPA design and size	MPA decision-making arrangements and fishers' participation	Fishery target species	Fishery tenure/management system	Number of fishers
**México** Bahía de Loreto National Park (NP) (1996)	Exclude industrial fisheries **Mixed**	Conservation, sustainable use	With zoning scheme Size: 2065.8 km^2^ (1820 km^2^ marine) 2 no-take zones (1.27 km^2^)	**Consultative management** through MPA management committee Fishers are influential in MPA decisions	Clams, sea cucumbers, finfish, scallops	**Predios (TURFs)** for protected species (e.g., sea cucumber) (1 year, renewable) **Fishing licenses** for other species (2–5 years, renewable)	~ 120
**México** Sian Ka’an Biosphere Reserve (BR) (1986)	Prevent excessive tourism development **Mixed**	Conservation, sustainable use	With zoning scheme Size: 5281 km^2^ (1531 are marine) 3 no-take zones (165 km^2^)	**Consultative management** through MPA management committee Fishers are influential in MPA decisions	Lobster and small amounts of finfish	**Concessions (TURFs)** granted to lobster cooperatives (20 years, renewable) Individual parcels allocated to fishers through customary rules	~ 100
**Colombia** Ciénaga de la Caimanera Regional Integrated Management District (RIMD) (2008)	Halt mangrove destruction due to road construction and illegal logging **Top-down**	Conservation, sustainable use	With zoning scheme Size: 21.25 km^2^ (1.86 km^2^ marine, and a lagoon whose main water body is 1.88 km^2^)	**Consultative management through informal means** Environmental agency has been supportive of community initiatives	Coastal lagoon and mangrove fishes	**Informal fishing rights** recognized by government agencies Fishers’ organizations have considerable autonomy to define and monitor regulations	~ 80
**Colombia** **Protected Area complex:** Corales del Rosario y San Bernardo National Natural Park (NNP) (1977) Archipiélagos del Rosario y San Bernardo MPA (2005)	Prevent environmental degradation due to population growth and tourism development **NNP: Top-down** **MPA: Mixed**	**NNP:** Conservation **MPA:** Conservation, sustainable use	The **MPA** (1200 km^2^) includes the **NNP** (5586 km^2^) within its boundaries	**From non-participatory to participatory management** Afro-descendant communities, initially excluded, now must participate in management of the NNP, and are represented in an MPA management committee created in 2022	Coral reef species: lobster, queen conch, white fish	**From informal to formalized fishing rights** Granting of land entitlements to afro-descendant communities triggered recognition of fishing rights as a traditional livelihood	~ 80
**Colombia** Golfo de Tribugá and Cabo Corrientes Regional Integrated Management District (RIMD) (2014)	Prevent environmental degradation (reduce industrial fisheries and massive tourism) **Bottom-up**	Conservation, sustainable use, protection of traditional populations’ culture and livelihoods	With zoning scheme Size: 601.38 km^2^ (~ 95% marine) Without no-take zones	**Consultative management** through MPA advisory committee Afro-descendant community councils and fishers are influential in RIMD decision making	Demersal and pelagic finfish, mangrove cockles, shrimps	**From informal to formalized fishing rights** Granting of land entitlements to afro-descendant communities triggered recognition of fishing rights as a traditional livelihood	~ 600
**Ecuador** Galápagos Marine Reserve (MR) (1998)	Prevent expansion of sea cucumber fishery, massive tourism, and industrial fisheries **Top-down**	Conservation, sustainable use	With zoning scheme Size: 146 598 km^2^ (138 872 km^2^ marine) 31 no-take zones (44 925 km^2^)	**Deliberative management until 2009** through two nested decision making bodies with stakeholders’ representation **2009-present:** While MPA authorities have generally consulted the fishing sector, the legally mandated advisory board has yet to be fully implemented	Sea cucumbers, lobsters	**Fishing licenses** Generalistic (all species) 2-yr license (renewable) Limited-entry: exclusive access rights for resident small-scale fishers	~ 400
**Ecuador** 6 de Julio Cooperative Mangrove Custodia (MC) (2000)	Halt mangrove destruction by shrimp farms **Bottom-up**	Conservation, sustainable use, securing ancestral groups' rights	Size: 20.36 km^2^ (all mangrove area, excludes coastal water)	**Community-based management** TURF holders responsible for protecting and managing custodias	Mangrove cockles and crabs	**Custodia (TURF)** Exclusive use-rights granted to organized ancestral communities or groups (10 years, renewable)	~ 150
**Brazil** Canavieiras Extractive Reserve (ER) (2006)	Halt mangrove destruction by shrimp farms, exclude industrial fisheries and massive tourism **Bottom-up**	Conservation, sustainable use, protection of traditional populations’ culture and livelihoods	With zoning scheme Size: 1000 km^2^ (83% marine, 17% terrestrial/ mangrove) Without no-take areas	**Deliberative management** through a council with majority representation of fishing communities (50% + 1 seats)	Finfish and shellfish species	**Exclusive and long-term use-rights** Granted to traditional populations (fishing communities) through a 20-yr use-contract (renewable)	~ 2500 beneficiaries (with fishing rights)
**Brazil** Arraial do Cabo Extractive Reserve (ER) (1997)	Exclude industrial fisheries, massive tourism, port and oil-related activities **Bottom-up**	Conservation, sustainable use, protection of traditional populations’ culture and livelihoods	Size: 516 km^2^ (only marine) Without no-take areas	**Deliberative management** through a council with majority representation of fishing communities (50% + 1 seats)	Shellfish, pelagic and migratory finfish	**Exclusive and long-term use-rights** Granted to traditional populations (fishing communities) through a 20-yr use-contract (renewable)	~ 2000 beneficiaries (~ 300 active fishers)
**Chile** Mar de Juan Fernández MUMPA (2016)	Exclude industrial fisheries **Bottom-up**	Conservation, sustainable use	With zoning scheme Size: 24 000 km^2^ (all marine) 5 no-take zones (1000 km^2^)	**Deliberative management** through a local council with stakeholders’ representation to co-manage the area	Lobsters, finfish species for bait and local consumption	**Customary sea tenure** Rules about ownership and transferability of discrete trap fishing spots	~ 120
**Argentina** Península Valdés Natural Protected Area (NPA) (2001)	Foment tourism and exclude industrial fisheries **Top-down**	Conservation, sustainable use	Unenforced zoning scheme Size: 9387 km^2^ (4653 km^2^ marine)	**Non-participatory management** Fishers not represented in the MPA decision-making body	Shellfish, mainly scallops	**Fishing licenses** Multispecific (1 year, renewable) Limited-entry system for scallops diving **Until 2009: consultative fisheries management** through technical committee with organized fishers representation	~ 150

We classified the case studies according to the initial approach to governance of the MPA (referred hereafter as MPA origin) into three categories (Table 1): (1) bottom-up, where local people had a substantial role in promoting and establishing the MPA (community led), (2) top-down, where state agencies had a predominant role (government led), and (3) mixed, where community and government had similar weight in driving the initiative.

We analyzed how the existence of the MPA affected various dimensions of SSFs governance, namely: (1) Interactions with competing uses, (2) access, tenure security and stewardship, (3) decision-making arrangements, (4) monitoring and research, (5) fisheries regulations, (6) enforcement and compliance, (7) fishers’ organization, (8) socioeconomic aspects, and (9) management capacity. For each dimension, we developed working hypotheses about expected positive/beneficial and negative/detrimental effects of the MPA (Table 2). For example, for the first dimension, positive effects might include improvements in: the definition of access rights, recognition of historical/legitimate users’ rights, security of tenure, and resource and environmental awareness/stewardship; negative effects might include increased intersectoral conflicts and disruption of traditional access mechanisms. The dimensions of fisheries governance and the possible hypothetical effects of MPAs on these dimensions were identified by the authors based on the literature and their own experience with fisheries and MPAs in numerous case studies in the LAC region.

**Table 2:** Hypothetical expected positive and negative effects of MPAs on dimensions of SSFs governance

SSF governance dimensions	Hypothetical expected positive effects of MPAs on SSF governance	Hypothetical expected negative effects of MPAs on SSF governance
Interactions with competing uses	Exclusion of industrial fleets (trawlers), other competing users (other SSFs communities), development threats (ports, oil exploration, real estate development), damaging fishing gears	Negative externalities of tourism or from other allowed activities
Access, tenure security and stewardship	Increased definition of access rights Increased recognition of historical/legitimate users´ rights Increased recognition of traditional/local populations´ rights (e.g., to maintain traditions, to self-determination) Increased security of tenure (refers to length and juridical security) Increased resource stewardship (and compliance with rules) Increased environmental awareness or stewardship	Increased social conflicts due to exclusion or legitimacy issues jeopardizing stewardship Debilitation/disruption of traditional access mechanisms/systems
Decision making arrangements	Establishment of collective decision-making bodies and enhanced institutions Increased participation and alliances (among NGOs, academia, users, and government agencies) Increased knowledge sharing (local/traditional & scientific) derived from participation	Competing interests/agendas favoring most powerful/influential sectors Exclusion from decision-making. Limited or ineffective participation
Monitoring and research	Increased monitoring Increased scientific research	Scientific monitoring/knowledge conflicts with traditional/local knowledge and management systems
Fisheries regulations	Increased fisheries (and other activities) regulation (improved planning and practices)	Increased conflict with traditional management Increased competition/conflict among and within groups of MPA users due to excessive/inadequate/inequitable restrictions established
Enforcement and compliance	Increased resources/personnel devoted to fisheries/MPA enforcement Increased collaborative efforts to make enforcement more efficient (e.g., interagency-community efforts)	Formal enforcement mechanisms conflict with informal/traditional management mechanisms The need to coordinate activities among several agencies challenges/reduces the effectiveness of enforcement efforts
Fishers' organization	Increased incentives for fishers to organize Increased community empowerment	Increased burden for fishers (i.e., responsibilities, time, money) because of participation and co-administration Debilitation of social cohesion/organization/leadership due to weak or inadequate government response
Socio-economic aspects	Increased opportunities for livelihoods diversification (e.g., ecotourism) Improved benefits derived from improved regulation/attention to fisheries issues	Exclusion/disruption of means of survival
Management capacity	Increased attention to fisheries issues (e.g., management, conflicts) Increased delegation of management authority to users Increased training and financial opportunities (e.g., participation facilitated and funding provided by International organizations)	Increased state presence and authority is a barrier for self-governance Increased state presence and authority conflicts with traditional management systems The need to coordinate activities among several agencies challenges/reduces the effectiveness of government actions Diminished management capacity due, for example, to increased conflict among users or international standards and targets not necessarily matching the local context

We evaluated whether the postulated effects were present in each of the case studies using four categories: (1) ‘observed’ (present); (2) ‘partially observed’ (partially present); (3) ‘not observed’ (not present); and (4) ‘not applicable or information not available.’ For example, if the establishment of the MPA led to clarification of access rights, the effect was considered ‘observed’ or ‘partially observed,’ depending on the salience of the effect. The scores were assigned using information on the entire MPA trajectory obtained from publications and based on our own experience in collaborative research initiatives and/or as members of technical fisheries/conservation committees for the areas. Authors who had the most direct experience with each case study assigned the scores. Where more than one co-author had first-hand knowledge of a particular case, they consulted and assigned the scores jointly by consensus.

A non-metric multidimensional scaling (nMDS) analysis was conducted to explore whether there were grouping patterns among MPAs based on the observed effects on SSF governance dimensions. Non-metric multidimensional scaling methods are useful for spatially representing the interrelationship among a set of data objects (Rabinowitz 1975). Based on the scores assigned in Tables 3 and 4, numerical values were given to each response category as follows: observed = 3, partially observed = 2, not observed = 1, and no information available or not applicable = 0. The free software PAST 4.0 was used.

**Table 3:**
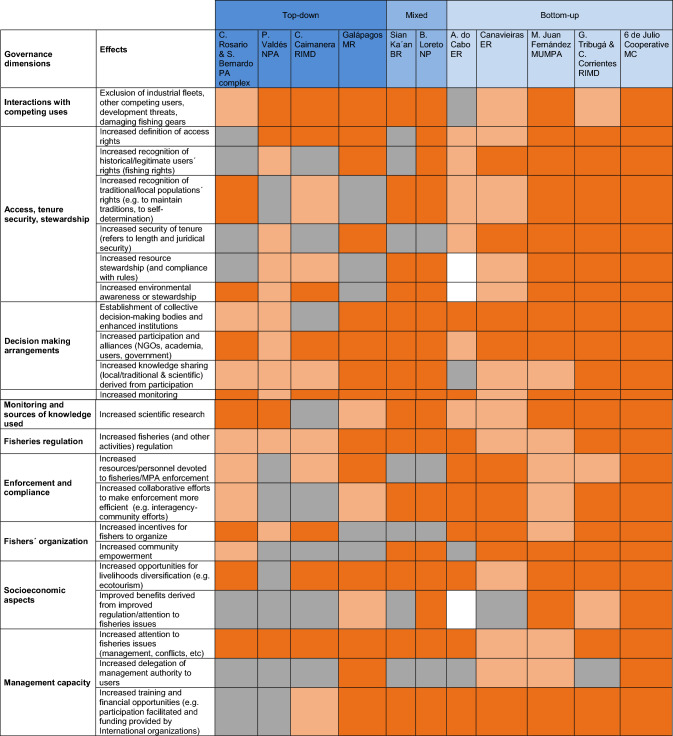
Observed positive effects of MPAs on relevant dimensions of SSFs governance

Colors indicate: dark orange = observed, light orange = partially observed, gray = not observed, white = no information available or not applicable to that case. For ease of visualization, case studies were arranged along a origin-type gradient from top-down (in blue on the left) to bottom-up on the right (pale blue), with mixed cases in the middle (light blue)

**Table 4:**
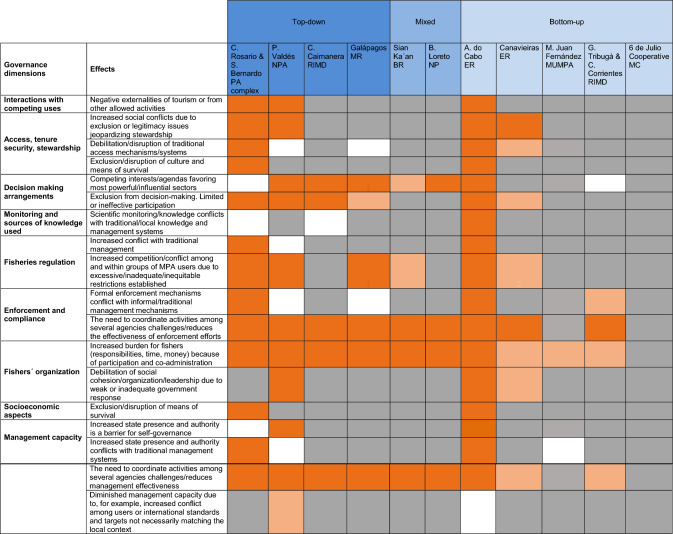
Observed negative effects of MPAs on relevant dimensions of SSFs governance

Colors indicate: dark orange = observed, light orange = partially observed, gray = not observed, white = no information or not applicable to that case

## Results

**Fig. 2:**
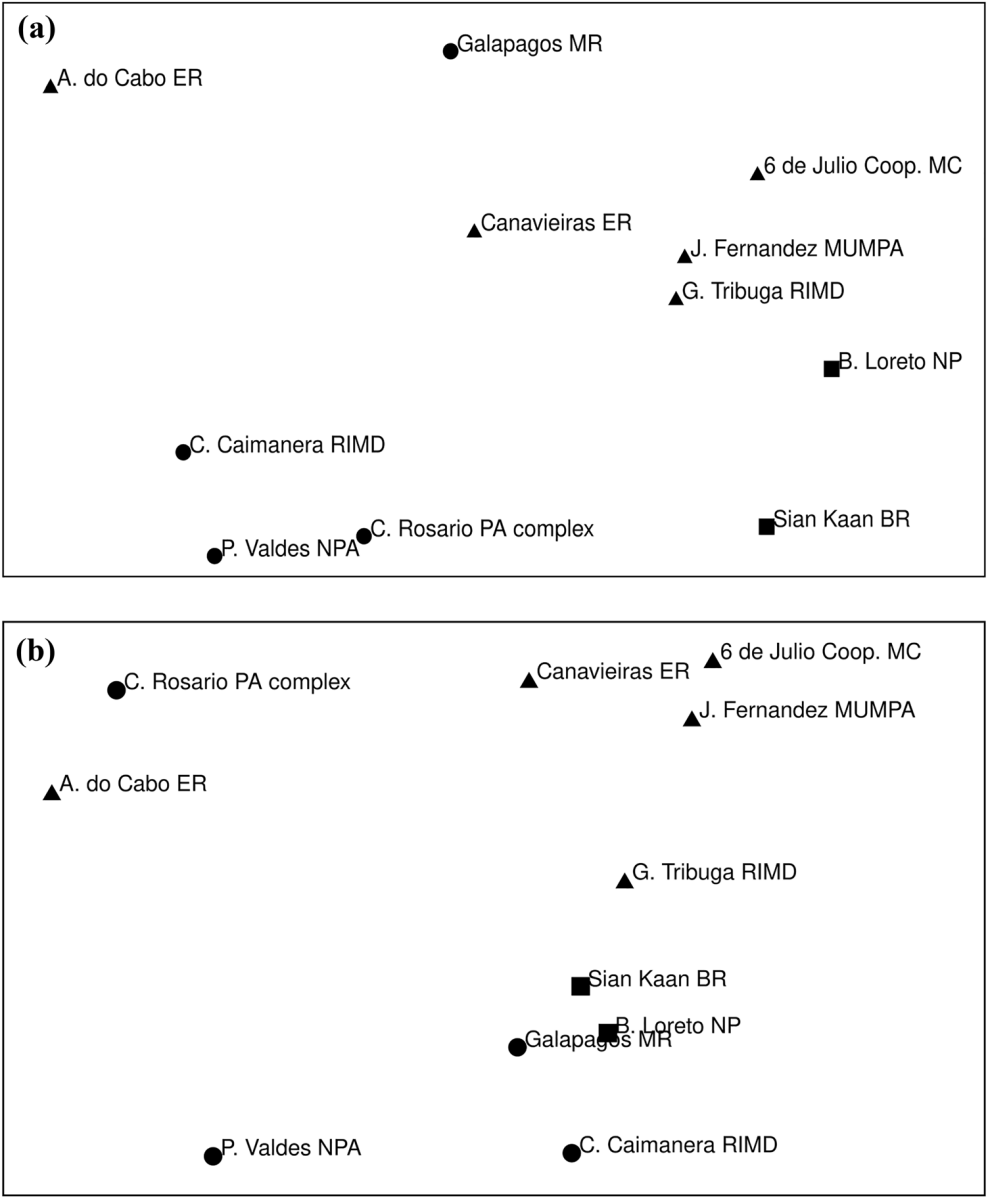
Multidimensional scaling output showing the grouping patterns of MPAs based on the observed. **a** Positive effects on SSF governance dimensions. **b** Negative effects on SSF governance dimensions. MPA names were shortened to ease visualization (Spanish accents not admitted by the software). Black dots indicate MPAs of top-down origin, black squares MPAs of mixed origin and black triangles MPAs of bottom-up origin

Overall, MPAs had many positive effects on SSF governance (Table 3) and relatively few negative effects (Table 4). Positive effects were more frequent or more pronounced in areas with mixed and bottom-up origin, with a few exceptions, while several negative effects were more frequent in areas with top-down origin, again with exceptions. Interestingly, some positive effects were found across the entire gradient of MPA origins, from top-down to bottom-up. This was the case, for example, of the exclusion of competing uses, improved access rights definition, greater attention to fisheries issues, increased monitoring and research, and higher opportunities for livelihoods diversification (Table 3). Likewise, a few negative effects were observed in most MPAs independently of their origin, for example, a reduction in the effectiveness of enforcement and management efforts due to poor coordination among management agencies, or an overburden from MPA-derived responsibilities (Table 4). The following sections describe the results for each of the dimensions of SSF governance examined (based on Tables 3 and 4), followed by a description of the multidimensional scaling results (based on Fig. 2a, b).

### Effects of the MPA on different dimensions of SSF governance

#### Interactions with competing uses

In all of the case studies, the establishment of MPAs contributed to reduce or completely exclude activities that were harmful to the environment and also affected SSF (Table 3).

Preventing the entry of industrial fishing vessels has been a major motivation for the creation of many of the MPAs reviewed. For example, the Bahía de Loreto National Park (NP) (mixed) aimed to reverse the environmental degradation caused by industrial trawling. In the Mar de Juan Fernández MUMPA (bottom-up), the community requested the creation of the area to exclude industrial vessels that fished around the Juan Fernández Archipelago (J. Chamorro, community leader, pers. comm. 2022). In Península Valdés, industrial dredging, which had caused the collapse of the scallop beds in a neighboring gulf, was banned in the mid-1970s through the creation of a Marine Park, a precursor to the Península Valdés Natural Protected Area (NPA) (top-down). In the Golfo de Tribugá and Cabo Corrientes Regional Integrated Management District (RIMD) (bottom-up), industrial fleet’s operations were significantly reduced (5 months a year in about half of the trawling grounds, Rueda et al. 2020) through an agreement with the artisanal sector that participates in the co-management of the area. In the Galápagos Marine Reserve (MR) (top-down), resident small-scale fishers were granted exclusive access rights, resulting in the exclusion of the industrial fleet from the reserve. In all these cases, the imposed restrictions have been effective. In the Arraial do Cabo Extractive Reserve (ER) (bottom-up), while industrial fishing was excluded (although not fully enforced), other potentially harmful uses persist: a port, a pier, mooring space for numerous vessels, large-scale tourism and naval facilities. There is also intensive oil-related activity in the surrounding marine area and an oil spill was registered in a beach of the reserve (Porto do Forno) in 2019.

The creation of MPAs has also prevented excessive tourism development in some cases, for example, in Quintana Roo, where the Sian Ka’an Biosphere Reserve (BR) (mixed) has preserved the way of life of fishing villages fostering low-impact tourism (Méndez-Medina et al. 2015). In other cases, unregulated tourism has negatively affected the areas and local fisheries. For example, in the Peninsula Valdés NPA and the Corales del Rosario y San Bernardo protected area complex (see Table 1), both with top-down origin, tourism has been promoted for economic reasons, disregarding its negative impacts. In Península Valdés, visitors extract marine resources (e.g., fish, mollusks) without authorization, often commercializing the catches, disturbing the intertidal, and affecting the activity of authorized artisanal fishers. In the Arraial do Cabo ER (bottom-up), tourism activities have displaced artisanal fishers from traditional coastal zones because, according to fishers, tourists ‘drive away the shoals’.

Halting mangrove destruction due to shrimp farming, urban expansion, road construction or illegal logging for timber was also achieved through the MPAs. In the Ciénaga de la Caimanera Regional Integrated Management District (RIMD) (top-down), the area recovered from massive mangrove harvesting and a road construction that caused mangrove and fish mortality in the 1980s (Taver-Escobar et al. 2004). In Canavieiras Extractive Reserve (ER) (bottom-up), the reserve effectively stopped the expansion of shrimp farming which had destroyed mangroves and crab harvesting sites. Similarly, mangrove deforestation was significantly reduced in the 6 de Julio Cooperative Mangrove Custodia (MC) (bottom-up).

#### Access, tenure security and stewardship

In many of the cases, the establishment of the MPA led to improved clarity of harvest rights within the area, recognition of historical use rights (as fishers), recognition of traditional/local population rights (beyond fishing), enhancement of tenure^4^ security (duration and juridical security) and resources/environmental stewardship. Several of the positive effects were observed in many MPAs with mixed to bottom-up origin and, to a lesser extent, in MPAs with top-down origin.

Changes have been substantial in areas aimed at safeguarding the livelihoods and cultures of traditional/ancestral^5^ populations, such as the Extractive Reserves (ER) and Mangrove Custodias (MC) (both bottom-up) (Bravo 2013; Nobre and Schiavetti 2013; Cardozo et al. 2019). Detailed access rules and long-term use-rights are granted, for 20 years in ERs and 10 years in MCs. Implementation has been challenging though, particularly in the ERs case studies (as described later).

In some cases, access rights (considering historical use and traditional practices) had been recognized long before the MPA was established, but the MPA provided an additional institutional layer to secure those rights. For example, the lobster fishery of the Sian Ka’an BR (mixed) occurs within TURFs^6^ that were granted to cooperatives in the early 1990s for 20 years. The TURFs of three cooperatives are fully included in the reserve, while those of two other cooperatives are only partially included (CONANP 2014). Although the reserve was formalized in 1986, before the granting of TURFs, the cooperatives had developed informal rules since their early days (late 1960s) to allocate subdivisions of the marine area to members for the deployment of artificial lobster shelters (Méndez-Medina et al. 2015). Self-imposed rules are highly respected by cooperative members (e.g., entry is closed except for sons of fishers) (Orensanz et al. 2013; Méndez-Medina et al. 2020) and they are recognized by reserve authorities (Méndez-Medina et al. 2015).

The Juan Fernández lobster fishery also has a customary tenure system that has been in place for over a century (Ernst et al. 2010). Each fisher “owns” a number of fishing spots where lobster traps are deployed; the use and transfer of rights over spots are regulated by well-established internal rules which have limited the increase in fishing effort (Orensanz et al. 2013). The tenure system is now recognized by the government and fully contained within the Mar de Juan Fernández MUMPA established in 2016 upon fishers’ request to protect their livelihoods and the biodiversity of the region (J. Chamorro, community leader, pers. comm. 2022).

In the Golfo de Tribugá and Cabo Corrientes RIMD (bottom-up) the communities that fish inside the area were granted collective ownership of the land.^7^ The title does not include the sea, but communities hold the right to maintain traditional livelihoods like fishing (Roldán 2013; CODECHOCO et al. 2014). To claim territorial rights, communities are required to self-organize in Community Councils, which define entry and use rules (Orensanz et al. 2013). Although land ownership was fundamental to advancing community rights, the RIMD helped strengthen rights over marine resources. An agreement was signed between small and large-scale fishers to resolve historical conflicts and consolidate access rights for the artisanal sector. The agreement became law in 2018 and has an active follow-up committee (Rueda et al. 2020).

In MPAs with top-down origin like the Galápagos MR and Peninsula Valdés NPA, where no customary systems existed and fishers’ organizations were not as strong, the issuance of fishing licenses (albeit short-term) and the implementation of limited entry systems for specific resources helped to define access rights and recognized historical users. The achievement of sustainable harvests has been a greater challenge in these cases.

In the Galápagos MR, a moratorium on the registration of new fishers and exclusive access rights for resident fishers were introduced to regulate sea cucumber extraction. These measures were insufficient to prevent the collapse of the fishery in the mid-2000s. Multiple factors contributed, including weak fishers’ organization, a generalistic license system (not species-specific), a pervasive partnership between fishers and intermediaries (high demand from Asia raised prices exponentially) and insufficient control (Orensanz et al. 2013; Villasante et al. 2022). A participatory process to reform the fishing regulations, including the license system, is underway (as of first half of 2024).

In the Península Valdés NPA, a limited-entry system was introduced in the scallop diving fishery in 2001 in response to fishers’ requests that permits be exclusively granted to the 20 boat-owners with a history in the fishery. Lack of enforcement of exit rules resulted in a rigid (without renewal) and to some extent illegitimate system. Infractions are rarely punished and no permits have been withdrawn after non-compliance with the rules. Crew members with a long history in the fishery have hardly been able to obtain permits. Fisheries and conservation matters are managed by separate agencies, without coordination (Orensanz et al. 2013; Esteves et al. 2020). MPA authorities have been scarcely involved in fisheries management.

Among the observed negative impacts related to access, conflicts within or between sectors due to exclusion or legitimacy issues increased in some top-down areas, but also in some bottom-up areas where fisheries coexist with more powerful sectors.

For example, the designation of Península Valdés as a Natural World Heritage site in the mid-1990s led fishers to constitute a formal association to defend their livelihoods. Although diving for scallops was developed in the mid-1970s as a habitat-friendly alternative to dredging (effectively banned since 1974), fishing, even if small-scale and selective, has been perceived as a threat to conservation by MPA authorities and other influential sectors (landowners, NGOs). Around 90% of the land of Península Valdés is privately owned and used for sheep ranching (large extensions in a few hands), which has led to ongoing conflicts with fishers mainly around the restriction of access to the coast (Piñeiro 2021). The lack of fishers’ representation in the MPA Directorate exacerbates these disputes. The fishers still struggle to be recognized as legitimate users and to get authorities to address long-standing problems (e.g., illegal fishing, blocking of access).

Similarly, when the Corales del Rosario y San Bernardo National Natural Park (NNP) was established in 1977, fishing and customary management practices were disregarded due to the Park’s no-take approach, aimed at protecting the largest coral reef in the Colombian Caribbean. However, fishing continued in the area due to lack of alternative employment, high dependence on marine resources for subsistence, and limited enforcement capacity. The granting of collective land ownership to the Orika afro-descendant community in 2014 (see footnote 7), which recognized fishing as ancestral livelihood, together with the creation of the Archipiélagos del Rosario y San Bernardo MPA (of multiple-use) in 2005, which includes the Park within its boundaries, strengthened fishing families’ rights to use and decide over the marine area. As in the Golfo de Tribugá and Cabo Corrientes case, the granting of communal land titles adjacent to fishing territories was paramount to the protection of fundamental human rights.

In some MPAs with bottom-up origin where fisheries coexist with more powerful sectors, conflicts over ‘who will benefit’ increased between sectors with contrasting interests. For example, before the Canavieiras ER was created, fishing families mobilized in support of the reserve, clashing with tourism entrepreneurs and shrimp farmers who saw the ER as an impediment to their economic development. Due to the slow and bureaucratic administrative processes of ERs, many of the promised benefits have not yet materialized, and businessmen still lobby against the reserve.

In the Arraial do Cabo ER, cultural aspects are recognized by municipal authorities and in the management agreement, but it is the Deliberative Council—a highly asymmetrical arena in terms of power–who defines the criteria for inclusion as legitimate users of the reserve (the beneficiary profile). Conflicts increased with the enactment of the SNUC Law (Law 9.985/2000), which opened the door to non-fishing sectors as beneficiaries. Representatives of tourism operators and the port's administration were given a seat (with right to vote) at the Council. Power imbalances between the artisanal and tourism sectors make it difficult to reach agreements on decisions that could benefit the artisanal sector, which has hampered the protection of traditional populations’ rights.

#### Decision-making arrangements

Collective decision-making bodies were established in most of the case studies, increasing the participation of various non-governmental sectors in MPA management.

In MPAs with mixed and bottom-up origin, the decision-making approach has been inclusive from the outset. In some cases, fishers already enjoyed a high degree of autonomy before the MPAs were established. In Bahía de Loreto NP and Sian Ka’an BR (both mixed) fishing organizations play an advisory role together with tourism, science, and civil society representatives. Although these are consultative councils, fishers still have significant influence on decisions.

In the Mar de Juan Fernández MUMPA (bottom-up), a participatory process has been completed to draft a Management Plan; the plan specifies that a Local Council with community and government representatives will co-manage the archipelago’s MPAs. Community representatives of three fishers' organizations, the tourism sector, the sport fishing sector, and a women's organization will integrate the Council. Similarly, in ERs (bottom-up), fishing communities have majority representation (half + 1 seat) in a Deliberative Council whose decisions are binding. In practice, however, government agencies play a dominant role, resembling a government-led MPA. The Environment Agency chairs the meetings, and decisions have to go through long and bureaucratic administrative processes at the federal level. In the Arraial do Cabo ER, the management plan was approved 23 years after its creation (MMA-ICMBio 2020). These long delays undermine trust, discourage participation and reduce the autonomy of fishing communities.

In the Golfo de Tribugá and Cabo Corrientes RIMD and the 6 the Julio Cooperative MC (both bottom-up), fishing communities make decisions more autonomously in local councils or assemblies. In the first case, community members respect the decisions made by the authorities, but they often propose changes, which are generally accepted. For example, when the central government applies a nationwide or Pacific-wide fishing restriction, the General Council often prepares stronger proposals with technical support from its partners (research institutes, NGOs). In the second case, Custodia members cannot make decisions on mangrove use that are not specified in the management plan (developed by fishers with technical assistance), but they can define the operational rules and day-to-day decisions to implement the plan. They can also add harvest measures (e.g., closing areas within their concession) without contradicting national norms and decide how to monitor locally, reporting infringements to the authorities. In both cases, state authorities intervene, but in a supportive role.

In the MPAs with top-down origin, the authorities were less inclined to allow participation and self-governance, at least initially, but some became more inclusive over time. For example, in the Corales del Rosario y San Bernardo NNP, organized communities gained a place in decision-making through formalized co-management (MADS 2020), while in the Ciénaga de la Caimanera RIMD some community organizations have been able to advice on planning and management through informal mechanisms, despite the fact that NNPs and RIMDs were not originally participatory figures (Ramirez 2016). Afro-descendant communities are also represented in the inter-institutional committee created in 2022 to manage the Archipiélagos del Rosario y San Bernardo MPA (MADS 2022). In the Península Valdés NPA, the fishing sector never had a seat at the Directorate, which represents landowners, businesses and tourism operators, in addition to the government. However, a proposal to incorporate a fishers’ representative is being considered as part of an ongoing revision of the NPA management plan (as of first half of 2024).

Other MPAs have instead become less inclusive over time. The Galápagos MR (top-down) initially embraced a co-management approach, where diverse stakeholders participated in a local and a national decision-making body, with small-scale fishers’ representation (also tourism, naturalist guides, NGOs, government authorities) (Heylings and Bravo 2007; Orensanz et al. 2013). Co-management stemmed from conflicts rooted in the historical exclusionary conservation efforts (since the 1930s) (Burbano et al. 2020). However, post-2008 legislative changes have significantly curtailed fishers’ participation (Llerena et al. 2015; Burbano et al. 2020; Castrejón et al. 2024) and a non-binding advisory board has been created by law, but its implementation is pending (as of April 2024). The fishing sector frequently finds itself sidelined, while government agencies, conservationists, and the tourism industry assert dominance, even during periods when participatory approaches have been emphasized (Heylings and Cruz 1998; Burbano et al. 2020).

Pronounced power imbalances among decision-making stakeholders, such as described for the Arraial do Cabo ER, the Península Valdés NPA and the Galápagos MR, have undermined the fishing sector's right to participation.

Where fishers’ participation in MPA decisions has been effective, the use of fishers’ knowledge in decision-making and the formation of alliances that facilitate better and faster responses to problems and conflicts have often been observed (see Table 3).

#### Monitoring and research

The establishment of MPAs has frequently fostered scientific research and monitoring of the impact of extractive and non-extractive activities on the conservation status of the MPA (Table 3).

In some cases, fisheries monitoring programs have been carried out with the active participation of local fishers, whose empirical knowledge has been incorporated into management decisions. In the Golfo de Tribugá and Cabo Corrientes RIMD (bottom-up), landings are monitored (officially required) by trained community members who also advise on the sampling design (e.g., site selection) (Rueda et al. 2020; Marco et al. 2021). Fishers are also involved in the design of tuna (*Thunnus spp.*) and red snapper (*Brotula clarkae*) fisheries monitoring surveys, advising on appropriate gear and location of breeding aggregation sites (Rodriguez et al. 2020). In the 6 de Julio Cooperative MC (bottom-up), scientific and fishers’ knowledge was used to design a monitoring program for the red crab (*Ucides occidentalis*) fishery in the Gulf of Guayaquil, where fishers participate in data collection (Cedeño et al. 2012a, 2012b).

In areas with enduring traditional fishing systems, fishers’ knowledge has informed decisions long before the MPA creation and continues to have a prominent role. In the Juan Fernández lobster fishery, the first locally crafted management measures were applied in the 1890s (J. Chamorro, community leader, pers. comm., 2022). A voluntary logbook program was launched in 2006 as a collaboration between the fishing community and scientists (Ernst et al. 2010), and it is still active to date (J. Chamorro, community leader, pers. comm., 2022). Although the MUMPA (bottom-up) did not have an appreciable impact on fisheries monitoring, it has fomented the initiation of a biodiversity monitoring program. Similarly, in the Sian Ka’an BR (mixed), most fisheries research and surveys are done collaboratively with the fishers. Recently, their knowledge of grouper and snapper breeding aggregations was used to design and implement a collaborative monitoring program, whose results were used to create fishing closures with community support (Fulton et al. 2018).

In the ER case studies (bottom-up), the enactment of the SNUC law (Law 9.985/2000) shifted the focus of the information used for reserve management to scientific knowledge, abandoning the central role that fishers’ empirical knowledge had in the early stages of ER implementation (see Lobão 2010). Furthermore, although scientific studies have increased since the establishment of both Canavieiras and Arraial do Cabo ERs, the information gathered has not been used meaningfully in fisheries or reserve management (Erler et al. 2015; B. Soares, Arraial do Cabo ER environmental analyst, pers. comm., March 2023). In addition, monitoring of fishing and other extractive activities and of conservation indicators has been sparse and intermittent.

On the other hand, in MPAs with top-down origin, collaborative efforts in fisheries monitoring have occasionally emerged. One example is the scallop diving fishery in the Península Valdés NPA, where collaborative surveys (between fishers, researchers and government staff) were implemented to assess stock status between 2001 and 2017. Fishers were key participants and their knowledge was directly applied to management (Dias et al. 2020). It is noteworthy that, for the most part, NPA authorities were not involved in this initiative.

#### Fisheries regulations

In most cases, MPAs have contributed to strengthening fisheries regulation across diverse institutional designs and MPA origins (Table 3). The legal obligations entailed by their creation have catalyzed the development of management plans and norms to reduce environmental degradation.

When pre-existing customary harvest rules were in place, these rules were in most cases recognized and adopted by MPA authorities (e.g., Sian Ka'an BR, Golfo de Tribugá and Cabo Corrientes RIMD, Corales del Rosario y San Bernardo protected area complex, Ciénaga de la Caimanera RIMD, Mar de Juan Fernández MUMPA).

In a few cases, new regulations introduced by MPA authorities disrupted pre-existing local arrangements. For example, in the Sian Ka'an BR (mixed), environmental authorities of Mexico City unilaterally created a no-take zone (Cayo Culebras) in Ascensión Bay, which overlapped with historic lobster fishing grounds and was neither respected nor enforced. In others, gear restrictions imposed by MPA authorities have been considered excessive by the fishing sector, exacerbating conflicts. For example, longline fishing for tunas was banned in 2000 in the Galapagos MR (top-down) to prevent illegal catch of sharks and other vulnerable species. Although several studies have assessed the impact of longlines on protected species, studies are not conclusive due to methodological differences that preclude reliable comparisons; this has affected the legitimacy of the ban, reducing compliance (Castrejón and Defeo 2023). The ongoing participatory process to reform fishing regulations is expected to address the longline fishery issues (Castrejón et al. 2024).

#### Enforcement and compliance

The establishment of an MPA has frequently led to the allocation of additional financial and human resources to enforcement (Table 3). Nevertheless, increases in funding have often been insufficient to cover the high costs of effective enforcement programs.

In light of this, collaborative efforts emerged in several MPAs with mixed and bottom-up origin. Within the Sian Ka'an BR (mixed), both formal and informal initiatives unfolded due to the limited presence of the fisheries agency (CONAPESCA) (Méndez-Medina et al. 2020). The northern reserve's surveillance committee exemplifies an effective collaboration among the environmental agency, lobster fishing cooperatives, the navy, and civil society organizations (*Ibid.*). This collaboration, enabled by strong fishers’ organizations, authorities who supported cooperative’s rules and proposals, and cooperation among several entities, capitalized on pre-existing informal arrangements between the cooperatives and the Navy, and garnered federal program support in the 2010s (*Ibid.*). The Bahía de Loreto NP (mixed) and the 6 de Julio Cooperative MC (bottom-up) also benefited from governmental programs (PROVICOM and Socio Bosque,^8^ respectively), enhancing their enforcement capabilities.

Similarly, the Golfo de Tribugá and Cabo Corrientes RIMD (bottom-up) brought institutional presence to the area as a direct result of proactive community efforts to provide office space for permanently hosting environmental and fisheries authorities at the General Council’s headquarters, where nine of the region's community councils are represented. This RIMD is effectively safeguarded by both government staff and community members (L. Perea, community leader, pers. comm., May 2021), exemplifying the pivotal role of robust community organizations.

In other areas, community members guarded their fishing territories long before the establishment of the MPA, prompted by the absence of fisheries authorities, and the need to resolve resource-use conflicts and protect customary or formal rights. In the Mar de Juan Fernández MUMPA (bottom-up), the community enforces its internal rules and has the support of the navy (in the islands, for sovereignty reasons) for radar detection of foreign fleets. Local fishers and navy personnel are in close contact (J. Chamorro, community leader, pers. comm., 2022). Similarly, in the Ciénaga de la Caimanera RIMD (top-down) local fishers stepped in due to the low capacity of the fisheries authority (AUNAP). Although the environmental authority (CARSUCRE) that manages the RIMD also has limited enforcement capacity, it has actively supported community conservation activities. An enclosed fishery with a unique entrance, a small and close-knit community with well-intentioned leaders and supportive authorities have contributed to this outcome (Saavedra et al. 2015; Ramirez 2017).

In contrast, in some MPAs with top-down origin, such as the Península Valdés NPA, community engagement in enforcement has been discouraged, despite the fact that government agencies lack the capacity for effective enforcement (Orensanz et al. 2013; Esteves et al. 2020).

In addition, several MPAs with significant government intervention (top-down and mixed, and a few bottom-up, Table 4) have suffered from inadequate state support and poor coordination between fisheries and environmental agencies with fragmented responsibilities, ostensibly impacting enforcement. However, it is worth noting that when fishing groups/communities have been allowed and supported to participate in enforcement, the results have generally been positive.

#### Fishers’ organization

In most cases, the establishment of an MPA has catalyzed the formation or strengthening of fishers’ organizations (Table 3). In MPAs with top-down origin, organization emerged as a defensive response prompted by fishers’ fears of being excluded from the area, such as in the Península Valdés NPA and the Corales del Rosario y San Bernardo NNP.

The establishment of formal organizations is often a prerequisite for stakeholders to participate in MPA and fisheries institutional processes (e.g., in developing management plans), and to apply for government benefits directed at the fishing sector. Formalization is also a way to bring the demands of the fishing sector to the attention of the authorities.

In most of the MPAs with mixed and bottom-up origin, the establishment of the MPA has empowered the fishing sector and increased its social cohesion (Table 3). In several of these cases (e.g., Sian Ka’an BR, Mar de Juan Fernández MUMPA, Golfo de Tribugá and Cabo Corrientes RIMD, Canavieiras ER), strong community organizations predated the MPA, moved by the urgency to meet basic needs (such as housing, drinking water and health care) and to defend their fishing grounds in remote, hard-to-reach areas with little or no government presence. Such strong organizations facilitated their involvement in the MPA institutional processes where they successfully channeled their demands. Similarly, in the Ciénaga de la Caimanera RIMD (top-down), community organization began in the 1980s, supported by a fisheries official who wanted to facilitate members’ access to government benefits (i.e., fishing equipment) (Ramirez 2017). Their organization grew stronger over the years, motivated by the low government presence for enforcement. The RIMD was established in the 2000s, and the environmental agency in charge has respected the community’s right to organize and create local committees (to promote tourism, protect mangroves, empower women) to improve the livelihoods and well-being of its members.

In other cases with less organizational history, such as the 6 de Julio Cooperative MC (bottom-up), the allocation of use rights over mangroves incentivized organization (formalization is required to apply for the concession). Fishers distributed roles among members and developed rules of conduct and sanctions, leading to high levels of compliance and participation in meetings (Bravo 2006, 2013). Other Custodias have been shown to strengthen fishers’ organization, social cohesion, leadership and entrepreneurial capacity (Altamirano et al. 1998; Coello et al. 2008; Beitl 2011, 2017).

Among the negative impacts of the MPAs, an intensified administrative burden on fishers has been highlighted in most cases (Table 4). Leadership, social cohesion and organization in the fishing sector have also been weakened in some cases by excessive government delays or unsupportive authorities. Delays in administrative processes, disappointment from unmet expectations, and suspensions of institutional processes for political reasons have sometimes eroded trust in government agencies and drained organizational vigor. Examples include the Canavieiras and Arraial do Cabo ERs (bottom-up). Even though the Deliberative Council of Arraial do Cabo ER incentivized the fishing sector to organize and become formally represented, there was fragmentation in many associations and lack of rotation of fisher representatives. Similarly, in the Peninsula Valdes NPA (top-down), the fishers’ association has been weakened over the years by government inaction and the interruption of numerous institutional processes (e.g., three frustrated attempts to revise the NPA management plan) in which the fishers actively participated. Young fishers have recently taken the lead and are revitalizing the organization.

#### Socioeconomic aspects

The introduction of MPAs has provided opportunities for livelihood diversification in most cases (Table 3).

For example, ecotourism developed successfully in the Ciénaga de la Caimanera RIMD (top-down) and the Sian Ka'an BR (mixed). In the Ciénaga, tourism activities reduced pressure on fishing and mangrove harvesting and alleviated poverty (Ramirez 2016). Community members also receive economic compensation from government and private funds for their mangrove restoration efforts. Local committees have been instrumental in improving income-generating opportunities. In Sian Ka’an, the Punta Allen fishing village has a thriving tourist industry (snorkeling and dolphin-watching trips, fly-fishing), which is well regulated by reserve authorities (setting carrying capacity, allowable zones and permits). Several fishers have been trained as nature and fly-fishing guides and in Basic English. To preserve the biodiversity of the bay for tourism, the fishing cooperative renews fishing licenses for species they do not harvest (finfish, shark, crab), so that no one else can enter the lobster concession (community leader, pers. comm., 2017). Ecotourism has reduced pressure on the lobster fishery. The main barrier to increasing income from tourism is difficulty of access. In contrast, in the Arraial do Cabo ER (bottom-up), some fishers have started to run taxi boats for tourists, but the power of the larger tourism industry hinders the adoption of measures by the Deliberative Council to support and develop community-based tourism.

In other areas, like in the Custodia case study (bottom-up), the rights granted have secured the livelihoods of families dependent on mangrove fisheries. Crab gatherers were able to buy motorbikes and improve their houses, and the organization started to provide small loans for members in cases of illness. Some members organized to sell processed crab and shellfish meat, including an all-female group (Bravo 2013). Part of the support received through the Socio Bosque program is used to provide social assistance to members’ families.

#### Management capacity

In general, the designation of areas as protected areas has increased the visibility of the sites and local fisheries, and thus the chances of receiving institutional support and funding for administration, research, monitoring and training, among other things.

In MPAs with mixed and bottom-up origin, the network of actors that participate in management is broader and more diverse (civil society organizations, universities/research centers, government actors, user sectors) than in MPAs with top-down origin. This greater diversity has generally enriched the support received through collaborations directed not only to conservation or fisheries, but also to social, cultural and economic dimensions (e.g., in Sian Ka'an BR, Golfo de Tribugá and Cabo Corrientes RIMD, Canavieiras ER, Bahía de Loreto National NP, Mar de Juan Fernández MUMPA). Constructive alliances between different actors have formed in many cases. Conversely, in MPAs with top-down origin that rely heavily on under-resourced government agencies (particularly in the Península Valdés NPA), management capacity is very limited. Bringing more partners to the table has led to significant improvements in most cases.

Notwithstanding, inter-agency coordination—vertically and horizontally—has been a major management challenge particularly in MPAs with top-down and mixed origin, but also in some areas with bottom-up origin (Table 4). As illustration, in the Bahía de Loreto NP (mixed), the environmental agency in charge of the MPA (CONANP) has limited fisheries responsibilities. Fishery resources are managed by the fisheries agency (CONAPESCA), under a different Secretariat (i.e., SADER). However, harvest authorizations for the sea cucumber, because the species was listed as ‘protected’ by a national norm, are issued by the wildlife protection agency (DGVS), which is under the same Secretariat as CONANP (i.e., SEMARNAT). The result is a heavy administrative burden, which challenges the coordination efforts by those responsible. In contrast, the Galápagos MR (top-down) is overseen by a single agency, the Galápagos National Park Directorate (GNPD), which has both environmental and fisheries responsibilities. However, this singular dependency has not guaranteed an equal balance in the exercise of both functions. Local fishers believe that the GNPD leans heavily toward conservation, often at their expense (Burbano et al. 2020). They demand a role for the National Fisheries Agency in the assessment and management of Galápagos fisheries, believed to have a superior technical capacity and a production-oriented perspective that is more in line with their interests and needs (M. Castrejón, pers. comm., 2023).

### Multidimensional scaling results

#### Grouping of MPAs on the basis of observed positive effects

The resulting grouping of MPAs reinforces the findings of previous sections (Fig. 2a). Several MPAs with bottom-up and mixed origin clustered on the right-hand side of the graph, all showing positive effects in all or most of the SSF governance dimensions (i.e., 6 de Julio Cooperative MC, Golfo de Tribugá and Cabo Corrientes RIMD, Mar de Juan Fernandez MUMPA, Bahía de Loreto NP and Sian Ka'an BR). Sian Ka’an was a little farther away from this group, likely because many positive social attributes were present before the reserve was created (i.e., defined access rights, security of tenure, delegation of management authority, strong community organization). In such cases, the effects were scored as “not observed” because, although present, progress could not be attributed to the MPA. The Canavieiras ER was also peripheral, as it had numerous positive effects but several were less pronounced (light orange), while the Arraial do Cabo ER was the farthest bottom-up area because it had several weak positive effects, and a few absent effects (e.g., an ineffective exclusion of competing uses and no increase in community empowerment), which were evidenced in the rest of the areas with bottom-up/mixed origin.

Among the areas with top-down origin, the Galápagos MR was close to the group of MPAs with bottom-up/mixed origin with the highest number of observed positive effects (it had a few unobserved positive effects, for example, on the fishers' organization dimension), while the rest of the areas with top-down origin (i.e., Península Valdés NPA, Corales del Rosario y San Bernardo protected area complex, Ciénaga de la Caimanera RIMD) grouped farther away. These MPAs had less positive effects than the rest of the areas and shared some absent effects in, for example, access (e.g., no or weak effect on tenure security), decision making (e.g., no or recent establishment of collective decision making bodies and poor knowledge sharing), enforcement (e.g., weak external support, no or weak collaborative efforts), management capacity (e.g., no or weak authority delegation and training/financial opportunities), and community empowerment.

#### Grouping of MPAs on the basis of observed negative effects

A subgroup of areas clustered on the right side of the graph, consisting of MPAs with no or very few observed negative effects (i.e., 6 de Julio Cooperative MC, Mar de Juan Fernández MUMPA, Golfo de Tribugá and Cabo Corrientes RIMD) (Fig. 2b). This group was close to a subset of MPAs (two with mixed origin—Bahía de Loreto NP and Sian Ka'an BR—and two with top-down origin—Ciénaga de la Caimanera RIMD and Galápagos MR) that shared some negative effects, such as agendas influenced by more powerful sectors, uncoordinated actions between government agencies, and overburdening from MPA-derived responsibilities (uncoordinated actions and overburdening were also observed in other MPAs). Canavieiras ER had several negative effects, but they were weak (e.g., increased social conflict, uncoordinated and delayed response by government agencies). The remaining MPAs (two with top-down origin—P. Valdés NPA and Corales del Rosario y San Bernardo complex—and one with bottom-up origin—Arraial do Cabo ER) were farther away from the rest and showed numerous negative effects in most SSF dimensions. It is worth noting that although fishers’ participation increased since the creation of the MPAs in most MPAs (see Table 3), all MPAs with a top-down origin and ERs (bottom-up) had limited or ineffective participation.

## Discussion

Overall, the MPAs evaluated in this study have delivered significant benefits for small-scale fisheries governance. Several positive effects were found across the entire gradient of MPA origins, from top-down to bottom-up, although benefits were more numerous and/or pronounced in MPAs with mixed and bottom-up origin. Protected areas generally contributed to enhance: the definition of access rights, the regulation of fisheries and other uses, the formation of alliances among diverse actors, monitoring and scientific research, the attention given to fisheries issues, and opportunities for livelihood diversification. Most MPAs have also limited or excluded competing large-scale developments and threats (e.g., industrial fisheries, real estate). In addition to those benefits, MPAs with mixed and bottom-up origin have augmented or reinforced the recognition of traditional/local populations’ rights, tenure security through longer-term use-rights, resource stewardship and rule compliance, as well as community empowerment, social cohesion and local organization. Innovative collaborations for enforcement and collective decision-making arrangements that effectively promoted fishers’ participation emerged in several cases.

In some of the cases with mixed and bottom-up origin, however, some pre-existing community attributes that eventually were instrumental for creating the MPA had a superlative role in progressing the governance of their fisheries. These include the existence of cohesive, interconnected social groups which, from the early fishery stages, were exposed to adverse conditions, sometimes in remote areas with a low state presence. In isolation, they searched for autonomous solutions to their basic needs and gradually became more organized. In order to exploit their fishery resources and defend their place and livelihoods against various threats, they developed local rules that have been adapted over time.

In addition to those enabling social attributes, the presence of supportive government agencies that recognized their own limitations, respected the rights of local people and leveraged local capacities and initiatives was essential in several cases. In some of the case studies, even where communities were not as well organized, some characteristics of government actors were conducive to positive outcomes for both fisheries and the MPA. These characteristics include the flexibility of authorities to adapt formal rules to local circumstances and to reduce bureaucratic requirements. A willingness to learn from community members and other actors, and to involve them meaningfully in institutional MPA processes, was important, even when fishers’ participation was not required or was even opposed by more powerful sectors. Openness to discuss and apply innovative solutions, which might fill regulatory vacuums or even conflict with existing legal frameworks (e.g., allowing community support for enforcement), was another attribute of government actors that led to positive outcomes in some cases. The need for flexibility in MPA and natural resources management processes, together with a commitment to seeking solutions, openness to new ideas, and responsiveness has been emphasized (Ross et al. 2002; Ferse et al. 2010).

Similarly, in many cases, partnerships with other external actors, such as academia and civil society organizations (including NGOs), have been instrumental in contributing to fisheries sustainability, biodiversity conservation and community well-being, particularly where collaborations have been established (e.g., for research and monitoring, fisheries and MPA management, capacity building, fundraising). The ways in which communities are supported by different actors and their interrelationships contribute to outcomes is a fruitful area for future research through social network analysis (Bodin and Prell 2011; Cárcamo et al. 2014).

The above conditions are consistent with a number of the critical enabling conditions for the sustainability of common-pool resources (CPRs) (e.g., fisheries, forests, water). According to CPR theory (Ostrom 1990; Agrawal et al. 2001), interdependence among group members (cohesiveness), shared interests and identities, shared norms, appropriate leadership, shared past successful experiences (social capital) are some of the key enabling characteristics of the group of people. Such conditions prevailed in several of our case studies that showed stronger social and governance attributes. Among the enabling conditions of the 'external environment' (sensu Agrawal 2001), the presence of supportive authorities that do not undermine the rights of users to design their own rules (or at least are designed with their active participation) (Design Principle 7 in Ostrom 1990; Agrawal 2001), long-term tenure rights to the resource granted to local users (also part of Ostrom’s Design Principle 7), and supportive external sanctioning institutions (e.g., for enforcement) (Agrawal 2001) have also fostered positive governance outcomes in several of our case studies. Although government support for enforcement is weak in most areas, in a few cases the authorities have received the support of local users to strengthen their enforcement capacity or have allowed the fishers to take a leading role in enforcement when authorities could not take that role. Certainly, several other factors contribute to the governance outcomes observed, as limiting conditions, for example, high resource demand and high degree of articulation with external markets (after Agrawal 2001) or as enabling factors, such as clear boundaries of resource users and resources, low mobility of target resources, or ecological attributes (after Ostrom 1990, 2009). The broader political setting might also have important limiting or enabling effects (*Ibid.*). We have focused on the social and institutional factors that facilitate adaptation to changing conditions (related to the flexible, collaborative approach mentioned above), favoring the resilience of the social-ecological system.

Our findings align with the literature on reconciling biodiversity conservation and fisheries management goals within an MPA context. For example, Weigel et al. (2014) analyzed numerous international MPAs (in Guinea-Bissau, Belize, the Philippines, Australia, the Mediterranean, Indonesia, Malaysia and Mozambique) and emphasized the need to integrate ecosystem conservation and resource management. The authors highlight the importance of creating spaces and processes for engagement, involving fishers in management, balancing costs and benefits to fishers, recognizing rights and tenure, coordinating among agencies and clarifying roles, combining no-take areas with other fisheries management measures, building a collaborative network, and managing adaptively. Bennett and Dearden (2014) identified similar contextual factors and inputs that have contributed to beneficial socio-economic and ecological outcomes in MPAs, highlighting the importance of clear, enabling and harmonized institutions (i.e., laws, policies, local norms); collaborative and coordinated networks, and participatory and contextualized implementation processes that focus on building trust. The authors emphasize that 'policies that support effective management and natural resource-dependent livelihoods include clear access rules and territorial rights, recognition of tenure, laws to support enforcement, legal mechanisms to support and ensure meaningful participation in design and implementation, and clarity of MPA objectives' (see various studies cited in Bennett and Dearden 2014). MPAs that exclude resource users from decision-making and ignore their rights and livelihoods can undermine their wellbeing and compliance (Grorud-Covert et al. 2021) and may constitute ocean grabbing (Aburto et al. 2020).

Lessons from MPAs around the world have shown that the underlying governance framework plays a critical role in the ability to effectively combine biodiversity conservation and resource use objectives (Kelleher 1999; Jones 2002; McCay and Jones 2011; Gaymer et al. 2014; Weigel et al. 2014; Bennett and Dearden 2014; Jones and Long 2021). Collaborative approaches (co-management) that integrate the merits of both top-down and bottom-up approaches and create synergies are generally recommended (*Ibid.*). Our findings based on MPA case studies from Latin America and the Caribbean region (LAC) provide further evidence of the benefits of combined approaches. Even the cases that we classified as bottom-up receive some level of external support, so they in fact combine both approaches.

A fully top-down approach to governance could theoretically be strictly enforced and effective, in places with strong government institutions, political will, and sufficient funding and management capacity (Weigel et al. 2014). For example, countries in North America, Australasia and Europe with relatively high governance capacity use government-led approaches to MPAs. This, however, does not mean that enforcement is solely from the top down; policies generally require full user involvement in MPA design, and participatory processes are often effectively implemented (Jones 2012). The vital role of local communities, among them indigenous groups, and other stakeholders in balancing conservation and resource use is now recognized also in developed countries (Armitage et al. 2017). In less favorable geopolitical contexts, such as in LAC, hierarchical approaches without substantial bottom-up support are doomed to fail, given limited management capacity associated with frequent economic crises and socio-economic unrest, high political instability (high turnover of officials), incomplete and weak legal frameworks, overlapping mandates and poor inter-agency coordination leading to implementation deficiencies, and inadequate budgets for MPA and fisheries governance. Government-led MPA participatory processes are oftentimes not truly participatory and suffer from power imbalances. Diverse stakeholder groups, including fishing communities, should be allowed and encouraged to participate in the establishment and management of MPAs, so that they can help design and implement collaboratively the many functions required for an effective governance that the state alone cannot guarantee. Given that the governance approach of an MPA is not set in stone, MPAs that currently have a highly hierarchical governance could significantly transform it to improve efficiency and equity if there is the political will and commitment to do so.

Our findings show that, in general, the MPAs analyzed generated numerous and diverse benefits for small-scale fisheries and their governance. Likewise, the fishing practices, values, knowledge, and initiatives of the artisanal fishing sector have contributed, in some cases in extraordinary ways, to the preservation of areas of vast ecological value. Local communities are partners in conservation, not aliens (Ferse et al. 2010), and must cease to be seen as a threat.

## Supplementary Information

Below is the link to the electronic supplementary material.

Supplementary file1 (pdf 955 kb)

## Footnotes

1 We use the term MPA as a generic term to refer to coastal and near-shore protected areas, regardless of how they are formally designated in each country or region.

2 Governance is about who decides what to do, how those decisions are taken, who holds power, authority, and responsibility, who is (or should be) held accountable (Hogg et al. 2019).

3 Parties to the Convention on Biological Diversity (CBD) adopted in 2010 Aichi Biodiversity Target 11, committing to conserve by 2020 at least 10 percent of coastal and marine areas through PAs and OECMs. That target was recently raised to 30 percent as part of 2030 Agenda (target 3, CBD 2022).

4 Tenure systems determine who can use what resources, for how long, and under what conditions (Charles 2011).

5 In ERs, traditional groups are defined as “culturally differentiated” groups that recognize themselves as such and have their own forms of social organization, that occupy and use territories and natural resources as a condition for their cultural, social, religious, ancestral and economic reproduction, using knowledge, innovations, and practices generated and transmitted by tradition’ (Decree 6040). In MCs, there is no official definition for ‘ancestral communities.’ They have been granted to local civil society organizations, fishing cooperatives and associations formally recognized by a governmental institution (Beitl 2011).

6 Stands for Territorial Use Rights in Fisheries (Christy 1982).

7 Law 70/1993 and Colombia’s 1991 Constitution recognize the right to collective ownership of historic territories occupied by Afro-Colombian communities, who make up around 90% of the Pacific coast population (Hoffmann 2007).

8 As reference, the 6 de Julio Cooperative MC received about USD 11,000/year in 2014 from this program to support enforcement (for three years, renewable).
