# Supplementary material for: Small-scale fisheries in ecologically sensitive areas in Latin America and the Caribbean: Do marine protected areas benefit fisheries governance?
Source: Ambio. 2024 Sep 6;54(1):20–42. doi: 10.1007/s13280-024-02062-z (PMC11607262; doi:10.1007/s13280-024-02062-z)
Supplement: Supplementary file 1 — Supplementary file1 (pdf 955 kb) [file 13280_2024_2062_MOESM1_ESM.pdf]

Article title: Small-scale fisheries in ecologically sensitive areas in Latin America and the Caribbean: Do Marine Protected Areas benefit fisheries governance?

Ambio: A Journal of environment and society

Authors: Ana Cinti, Luisa Ramirez, Mauricio Castrejón, Jaime A. Aburto, Luciana Loto, Stuart Fulton, Mario Rueda, Alexandre Schiavetti, Francisco J. Fernández-Rivera Melo, Manuel Bravo, Daniela Trigueirinho Alarcon, Valéria Penchel Araújo, Ana M. Parma

Corresponding author: Ana Cinti, Center for the Study of Marine Systems (CESIMAR), CCT CONICET-CENPAT. e-mail: cinti@cenpat-conicet.gob.ar.

Table S1: Basic attributes of case studies, arranged from North to South.

**Country/MPA (year created)**	**Trigger for MPA creation**	**MPA origin**	**MPA objectives**	**Primary conservation target**	**MPA design and size**	**MPA decision making arrangements (fishers' participation)**	**Target species**	**Fishery tenure/management system**	**Fishery fully or partially contained in MPA**	**Number of fishers**	**References used in review**
**México** Bahía de Loreto National Park (NP) (1996)	Exclude industrial fisheries	**Mixed.** Supported by the community, NGOs and government agencies	Conservation, sustainable use	Coastal habitat	With zoning scheme Size: 2065.8 km2 (1820 are marine) No-take zones: 2 (1.27 km2 total)	*** Consultative management** MPA Management Committee in which fishing organizations play an advisory role together with tourism, science, and civil society representatives Fishers are influential in MPA decisions	Clams, sea cucumbers, finfish, scallops	*** Predios (TURFs)** for species listed under special protection like the sea cucumber (for one year, renewable) * **Fishing licenses** for the rest of the resources (for 2-5 years, renewable)	Fully contained	~120	Ezcurra et al. 2002; Steinitz et al. 2005; Ramírez-Sánchez 2006; Avendaño-Ceceña 2007; Gerber and Salas 2007; Wielgus et al. 2007, 2008; Cudney-Bueno et al. 2009; Stamieszkin et al 2009; CCC 2010; Peterson 2010; Rife et al. 2013; CONANP 2019; Pellowe and Leslie 2020
**México** Sian Ka'an Biosphere Reserve (BR) (1986)	Prevent excessive tourism development	**Mixed.** Supported by the community, NGOs and government agencies	Conservation, sustainable use	Terrestrial, mangrove, and reef habitat	With zoning scheme Size: 5281 km2 (1531 are marine) No-take zones: 3 (165 km2 total)	*** Consultative management** MPA Management Committee in which fishers' organizations play an advisory role together with tourism, science, and civil society representatives Fishers are influential in MPA decisions	Lobster and a small amounts of finfish	*** Concessions (TURFs)** Cooperatives have exclusive rights to harvest lobsters via concessions granted for 20 years, renewable Fishers place artificial lobster shelters ("casitas") at individual parcels ("campos") which are allocated and transferred through customary rules	Concessions of three fishing cooperatives fully contained Concessions of other two cooperatives partially contained	~100	Seijo 1993; Sosa-Cordero and Ramírez-González 1993; Sosa Cordero et al. 2008; Orensanz et al. 2013; CONANP 2014; Ley-Cooper et al. 2014; Méndez-Medina et al. 2015, 2020; Fulton et al. 2018
**Colombia** Ciénaga de la Caimanera Regional Integrated Management District (RIMD) (2008)	Halt mangrove destruction due to road construction and illegal logging	**Top-down.**	Conservation, sustainable use	Mangrove habitat	With zoning scheme Size: 21.25 km2 (the lagoon's main water body is 1.88 km2)	*** Consultative management through informal means** Fishers not formally involved in RIMD's decisions But the responsible environmental agency has been receptive and supportive of	Coastal lagoon and mangrove fishes	*** Informal fishing rights** recognized by government agencies Fishing organizations have considerable autonomy in defining and monitoring fishing regulations due to limited capacity of the fisheries agency	Fully contained	~80	Taver-Escobar et al. 2004; Rueda et al. 2011; Saavedra et al. 2015; Delgadillo and Ruiz 2016; Ramírez 2016, 2017

						community initiatives					
**Colombia**<br>**Protected area complex:**<br>Corales del Rosario y San Bernardo National Natural Park (NNP)<br>(1977)<br><br>Archipiélagos del Rosario y San Bernardo Marine Protected Area (MPA)<br>(2005)	Prevent environmental degradation due to population growth and tourism development	**NNP: Top-down.**<br>**MPA: Mixed.**<br>With community support	**NNP:** Conservation<br>**MPA:** Conservation, sustainable use	Coral reef habitat	The MPA includes the NNP within its boundaries<br>**NNP:** with zoning scheme.<br>Size: 1200 km2 (practically all marine area, except for a few small islands and islets)<br>**MPA:** Zoning scheme to be defined.<br>Size: 5586 km2 (~90% is marine area)	*** From non-participatory to participatory management**<br>**NNP:** initially exclusionary. Nowadays afro-descendant communities that live in the islands must be involved in planning and management decisions (co-management contemplated in its management plan)<br><br>**MPA:** Afro-descendant communities are represented in the inter-institutional management committee created in 2022	Coral reef species: lobster, queen conch, white fish	*** From informal to formalized fishing rights**<br>The granting of land entitlements to afro-descendant communities triggered fishing rights recognition. The title does not include the sea, but communities hold the right to maintain traditional livelihoods such as fishing	Partially contained	~80	MMA-UAESPNN 2001; Durán 2009; Duque-Rico and Torres-Gómez 2011; INVEMAR-MADS 2012; Incoder-UJTL 2014; Ramírez 2016, 2017; MADS 2020, 2022
**Colombia**<br>Golfo de Tribugá and Cabo Corrientes Regional Integrated Management District (RIMD)<br>(2014)	Prevent environmental degradation (reduce industrial fisheries and massive tourism)	**Bottom-up.** With support from government agencies and other stakeholders	Conservation, sustainable use, protection of traditional populations' culture and livelihoods	Mangrove, estuarine, and coral reef habitat<br>Migratory corridor of humpback whale	With zoning scheme<br>Size: 601.38 km2 (~95% is marine area)<br>Without no-take zones	*** Consultative management**<br>Advisory committee of the MPA with representatives of environmental and fisheries authorities, NGOs, research institutes, afro-descendant communities and fishing cooperatives<br>Afro-descendant community councils and fishers are influential in RIMD decision making	Demersal and pelagic finfish, mangrove cockles, shrimps	*** From informal to formalized fishing rights**<br>The granting of land entitlements to afro-descendant communities triggered fishing rights recognition. The title does not include the sea, but communities hold the right to maintain traditional livelihoods such as fishing	Partially contained because of distribution patterns of target species of small-scale and industrial fisheries	~600	Rodríguez et al. 2012; Roldán 2013; CODECHOCO 2014; CODECHOCO et al. 2014; Díaz et al. 2016; Neira et al. 2016; Cuervo-Sánchez et al. 2018; Rueda et al. 2020; Marco et al. 2021

**Ecuador**<br>Galápagos Marine Reserve (MR)<br>(1998)	Prevent expansion of sea cucumber fishery, massive tourism, and industrial fisheries	**Top-down.** With support from international NGOs and cooperation agencies	Conservation and sustainable use	Endemisms	With zoning scheme<br>Size: 146 598 km² (138 872 km² are marine)<br>No-take zones: 31 (44 925 km2 total)	*** Deliberative management (until 2009)**<br>Two nested decision making bodies with stakeholders' representation (tourism, naturalist guides, NGOs, government authorities, small-scale fishers)<br><br>*** At present:**<br>Non-binding advisory board (pending implementation)	Sea cucumbers, lobsters	*** Fishing licenses**<br>Generalistic license (not species-specific, for 2 years, renewable)<br>Limited-entry: exclusive access rights for resident small-scale fishers	Fully contained	~400	Heylings and Cruz 1998; Heylings et al. 2002; Edgar et al. 2004; Heylings & Bravo 2007; Castrejón 2011; Hockings et al. 2012; Castrejón and Charles 2013; Orensanz et al. 2013; Castrejón et al. 2014; DPNG 2014; Castrejón and Defeo 2015; LLerena et al 2015; Burbano et al. 2020; Villasante et al. 2022; Castrejón and Defeo 2023; Castrejón et al. 2024
**Ecuador**<br>6 de Julio Cooperative Mangrove Custodia (MC)<br>(2000)	Halt mangrove destruction by shrimp farms	**Bottom up.** With support from NGOs and government agencies	Conservation, sustainable use, securing ancestral groups' rights	Mangrove habitat	Size: 20.36 km2 (all mangrove area, excludes coastal water)	*** Community-based management.**<br>TURF holders responsible for protecting and managing custodias<br>Use and access rules internally defined, in line with a management plan developed with technical assistance from external institutions	Mangrove cockles and crabs	*** Custodia (TURF)**<br>Exclusive use-rights granted to organized ancestral communities or groups (10-years, renewable)	Partially contained	~150	Bravo and Abarca 1995; Altamirano et al. 1998; Bravo 2000, 2006, 2013; Coello et al. 2008; Beitl 2011, 2012, 2017; Cedeño et al. 2012a, b
**Brasil**<br>Canavieiras Extractive Reserve (ER)<br>(2006)	Halt mangrove destruction by shrimp farms, exclude industrial fisheries and massive tourism	**Bottom-up.** With support from government agencies and universities	Conservation, sustainable use, protection of traditional populations' culture and livelihoods	Terrestrial and mangrove habitat	With zoning scheme<br>Size: 1000 km2<br>(83% marine, 17% terrestrial/ mangrove)<br><br>Without no-take areas	*** Deliberative management**<br>Deliberative Council with representatives of fishing communities (50% + 1 seats), governmental agencies, NGOs, recreational/tourism groups, others	Finfish and shellfish species	*** Exclusive and long-term use-rights**<br>Granted to traditional populations (fishing communities) through a use-contract (20 years, renewable) signed between the environmental agency and an umbrella fishers' association (Associação Mãe)	Fully contained	~2500 beneficiaries (with the right to harvest resources)	Erler et al. 2015; Dias et al. 2018; Dumith 2018; Cardozo et al. 2019

**Brasil**<br>Arraial do Cabo Extractive Reserve (ER)<br>(1997)	Exclude industrial fisheries, massive tourism, port and oil-related activities	**Bottom-up.** With support from government agencies and universities	Conservation, sustainable use, protection of traditional populations' culture and livelihoods	Marine (coastal) habitat (the area excludes land)	Size: 516 km2 (only marine)<br>Without no-take areas	*** Deliberative management**<br>Deliberative Council with representatives of fishing communities (50% + 1 seats), government agencies, NGOs, recreational/tourism groups, others	Shellfish, pelagic and migratory finfish	*** Exclusive and long-term use-rights**<br>Granted to traditional populations (fishing communities) through a use-contract (20 years, renewable) signed between the environmental agency and an umbrella fishers' association (Associação Mãe)	Fully contained	~ 2000 beneficiaries (~300 are active fishers)	Britto 1999; Lobão, 2010; Loto 2012; Maranhão 2012; Penchel-Araújo and Souza-Nicolau 2018
**Chile**<br>Mar de Juan Fernández MUMPA<br>(2016)	Exclude industrial fisheries	**Bottom-up.** With support from NGOs and government agencies	Conservation, sustainable use	Endemisms, diverse habitats	With zoning scheme<br>Size: 24 000 km2 (all marine area)<br>No-take zones: 5 (1000 km2)	***Deliberative management**<br>Management Plan (drafted through a participatory process) includes a Local Council with representatives from the community and government agencies to co-manage the area	Lobsters, some finfish species for bait and local consumption	**Customary sea tenure**<br>Defined by a complex set of rules about ownership and transferability of *marcas* (discrete fishing spots)	Fully contained	~ 120	Ernst et al. 2010; SUBPESCA 2014; Aburto et al. 2019; Aburto and Varas 2022
**Argentina**<br>Península Valdés Natural Protected Area (NPA)<br>(2001)	Foment tourism and exclude industrial fisheries	**Top-down.** With support from NGOs	Conservation, sustainable use	Charismatic fauna: whales, sea lions, terrestrial mammal	With zoning scheme<br>Size: 9387 km2 (4653 km2 are marine)<br>With intangible marine zones (though unenforced)	*** Non-participatory management**<br>MPA decision-making body includes representatives of landowners, businesses, tourism operators and government agencies, but not fishers	Shellfish species, mainly scallops	*** Fishing licenses**<br>Multispecific license (one year, renewable)<br>Limited-entry system for scallops diving<br>* **Until 2009: consultative fisheries management** through technical committee with organized fishers, researchers and staff from fisheries agency	Fully contained	~150	Cinti et al. 2003, 2011; Parma et al. 2003; Orensanz et al. 2003, 2005, 2007, 2013, 2015; Fiorda et al. 2013; Fiorda & Parma 2015; Soria et al. 2017; Esteves-Dias et al. 2020

References

Aburto, J., and K. Varas. 2022. Primera aproximación de una estructura de gobernanza para el co-manejo de las Áreas Marinas Protegidas de Juan Fernández. XLI Congreso de Ciencias del Mar, 23-27 mayo, Concepción, Chile. (in Spanish)

Aburto, J., K. Varas, and P. Bravo. 2019. Empowering and organizing the community of the Juan Fernández Archipelago to manage their marine protected areas. National Geographic Pristine Seas, Internal report.

Altamirano, M., M. Bravo, R. Elao, R. Noboa, and J. Rosero. 1998. Otorgación en custodia de un área de manglar a un grupo de usuarios en el Estero Saca Mano. Un ejemplo de conservación y uso sustentable para usuarios tradicionales. Programa de Manejo de Recursos Costeros, Technical Report, Guayaquil, Ecuador. (in Spanish)

Avendaño-Ceceña, L. 2007. Bases para el manejo de la pesquería del pepino de mar (Isostichopus fuscus) en Bahía de los Ángeles, Baja California, México. Masters Thesis. Ensenada, México: Universidad Autónoma de Baja California. (in Spanish). https://doi:10.57840/uabc-366

Beitl, C. 2011. Cockles in Custody: the role of common property arrangements in the ecological sustainability of mangrove fisheries on the Ecuadorian Costs. Journal of the Commons 5(2): 485-512.

Beilt, C. 2012. Beyond collective action: a multi-scale analysis of sustainability in the mangrove fishery commons of coastal Ecuador. PhD thesis. Athens, Georgia: The University of Georgia.

Beitl, C. 2017. Decentralized Mangrove Conservation and Territorial Use Rights in Ecuador's Mangrove-Associated Fisheries. Bulletin of Marine Science 93(1):117-136. http://doi:10.5343/bms.2015.1086

Bravo, M. 2000. Concesiones para el uso y custodia del Manglar – Componente de Gestión Ambiental en el Golfo de Guayaquil. Unidad de Coordinación del Proyecto PATRA–Litoral, Ministerio del Ambiente, Technical Report, Guayaquil, Ecuador. (in Spanish)

Bravo, M. 2006. Análisis de los Acuerdos de Usos Sustentable y Custodia del Manglar otorgados a Usuarios Tradicionales para la Protección y Custodia del Ecosistema Manglar-Estudios de Caso de las Concheras 18 de Octubre (Provincia Esmeraldas), Seis de Julio (Provincia Guayas) y Mariscadores Costa Rica (Provincia El Oro)". Informe de Consultoría Programa de Manejo de Recursos Costeros (PMRC), Guayaquil, Ecuador. (in Spanish)

Bravo, M. 2013. Alianza Público-privada para la Gestión de los Manglares del Ecuador: Los Acuerdos para el Uso Sustentable y Custodia. USAID Costas y Bosques Sostenibles, Technical Report, Guayaquil, Ecuador. (in Spanish)

Bravo, M., and N. Abarca. 1995. Cockle fishers of Ecuador, trapped between environment degradation and poverty. In The 9 th 1995 Conference on Coastal Zone extended abstracts, 209-210. Tampa, USA: American Society of Civil Engineers.

Britto, R.C.C. 1999. Modernidade e Tradição: construção da identidade social dos pescadores de Arraial do Cabo (RJ). Niterói: Editora da Universidade Federal Fluminense. (in Portuguese)

Burbano, D.V., T.C. Meredith, and M.E. Mulrennan. 2020. Exclusionary decision-making processes in marine governance: The rezoning plan for the protected areas of the 'iconic' Galapagos Islands, Ecuador. Ocean & Coastal Management 185: 105066. https://doi:10.1016/j.ocecoaman.2019.105066

Cardozo, L., D. Alarcon, S. Campiolo, and A. Schiavetti. 2019. Governança Ambiental e Percepção sobre Processos Participativos na Reserva Extrativista de Canavieiras, Bahia, Brasil. Desenvolvimento e Meio Ambiente 50: 170-191. http://doi:10.5380/dma.v50i0.58825

Castrejón, M. 2011. Co-manejo pesquero en la Reserva Marina de Galápagos: Tendencias, retos y perspectivas de cambio. D.F., México: Fundación Charles Darwin/Kanankil/Tinker. (in Spanish)

Castrejón, M., and A. Charles. 2013. Improving fisheries co-management through ecosystem-based spatial management: The Galapagos Marine Reserve. Marine Policy 38: 235-245. https://doi:10.1016/j.marpol.2012.05.040

Castrejón, M., and O. Defeo. 2015. Co-governance of small-scale shellfisheries in Latin America: Institutional adaptability to external drivers of change. In Interactive Governance for small-

scale Fisheries. Global Reflections, eds. R. Chuenpagdee R, and S. Jentoft, 605-625. MARE Publications Series 13. Springer Cham.

Castrejón, M., and O. Defeo. 2023. Reconsidering the longline ban in the Galapagos Marine Reserve. Marine Policy 151: 105589. https://doi:10.1016/j.marpol.2023.105589

Castrejón, M., O. Defeo, G. Reck, and A. Charles. 2014. Fishery science in Galapagos: From a resource-focused to a social-ecological systems approach. In The Galapagos Marine Reserve. Social and Ecological Interactions in the Galapagos Islands, eds. J. Denkinger, and L. Vinueza, 159–186. Springer Cham. https://doi:10.1007/978-3-319-02769-2_8

Castrejón, M., N. Moity, and A. Charles. 2024. The bumpy road to conservation: Challenges and opportunities in updating the Galapagos zoning system. Marine Policy 163: 106146. https://doi.org/10.1016/j.marpol.2024.106146

Cedeño, I., M. Bravo, F. Solano, M. Peña, and R. Zambrano. 2012a. Abundancia Relativa y Estructura de Tallas de Cangrejo Rojo de Manglar (Ucides occidentalis) en el Golfo de Guayaquil. Boletín Especial 3 (2). Guayaquil, Ecuador: Instituto Nacional de Pesca/USAID Costas y Bosques Sostenibles. (in Spanish)

Cedeño, I., and M. Bravo. 2012b. Protocolo de Muestreo Participativo: Capturas Comerciales del Cangrejo Rojo de Manglar (Ucides occidentalis) en el Golfo de Guayaquil. Boletín Especial 3 (1). Guayaquil, Ecuador: Instituto Nacional de Pesca/USAID Costas y Bosques Sostenibles. (in Spanish)

Centro de Colaboración Cívica (CCC). 2010. Caso de estudio: proceso de revisión del programa de manejo de Bahía de Loreto a través de la mirada del CCC. Technical Report. (in Spanish)

Cinti, A., A.M. Parma, and J. M. Orensanz. 2003. Seguimiento de la pesca de vieiras en el Golfo San José durante la Temporada 2002. Recomendaciones para el monitoreo de la pesquería. Technical Advisory Board for the Management of the Commercial Diving Fishery, Chubut Province, Technical Report 7, Puerto Madryn, Argentina. (in Spanish)

Cinti, A., J.M. Orensanz, and A.M. Parma. 2011. Informe final: Elaboración del Documento del Plan de Manejo para la Pesca Comercial de Mariscos Mediante Buceo en el golfo San José. Centro Nacional Patagónico (CENPAT-CONICET), Technical Report, Puerto Madryn, Chubut. (in Spanish)

CODECHOCO. 2014. Declara el Distrito Regional de Manejo Golfo de Tribugá-Cabo Corrientes. Acuerdo 011.

CODECHOCO, Los Riscales, GICPA, Alcaldía de Nuquí, and MarViva. 2014. Distrito regional de manejo integrado Golfo de Tribugá - Cabo Corrientes: Propuesta de declaratoria de una nueva área marina protegida. Documento Técnico. (in Spanish)

Coello, S., D. Vinueza, and R. Alemán. 2008. Evaluación del desempeño de los acuerdos de uso sustentable y custodia de manglar de la zona costera del Ecuador. Ministerio del Ambiente del Ecuador/ Conservación Internacional/UICN/Ecobiotec, Technical Report, Guayaquil, Ecuador. (in Spanish)

Comisión Nacional de Áreas Naturales Protegidas (CONANP). 2019. Programa de Manejo Parque Nacional Bahía de Loreto. Distrito Federal, México: CONANP. (in Spanish)

CONANP. 2014. Programa de Manejo Complejo Sian Ka'an: Reserva de la Biosfera Sian Ka'an, Área de Protección de Flora y Fauna Uaymil y Reserva de la Biosfera Arrecifes de Sian Ka'an. DF, México: CONANP. (in Spanish)

Cudney-Bueno, R., L. Bourillón, A. Sáenz-Arroyo, J. Torre-Cosio, P. Turk-Boyer, and W.W. Shaw. 2009. Governance and effects of marine reserves in the Gulf of California, Mexico. Ocean & Coastal Management 52: 207–218. https://doi:10.1016/j.ocecoaman.2008.12.005

Cuervo-Sánchez, R., J.H. Maldonado, and M. Rueda. 2018. Spillover from marine protected areas on the Pacific coast in Colombia: A bioeconomic modelling approach for shrimp fisheries. Marine Policy 88:182–188. https://doi:10.1016/j.marpol.2017.10.036

Delgadillo-G, O., and T. Ruíz-A. 2016. A bathymetric study to support management plans for La Caimanera coastal lagoon, Gulf of Morrosquillo, Colombia. Journal of Coastal Conservation 20: 237–244. https://doi:10.1007/s11852-016-0434-z

Dias, M., R. Gomes, S. Batista, S. Campiolo, and A. Schiavetti. 2018. Participação popular na criação de unidades de conservação marinha: o caso da Reserva Extrativista de Canavieiras. Revista Direito GV 14: 912-936. https://doi:10.1590/2317-6172201834.

Díaz, J.M., L. Guillot, and M.C. Velandia. 2016. La pesca artesanal en la costa norte del Pacífico colombiano: un horizonte ambivalente. Bogotá: Fundación MarViva.

Dirección del Parque Nacional Galápagos (DPNG). 2014. Plan de manejo de las áreas protegidas de Galápagos para el buen vivir. Puerto Ayora, Galápagos, Ecuador: Dirección del Parque Nacional Galápagos. (in Spanish)

Dumith, R.D.C. 2018. Dez anos de r-existência da Reserva Extrativista de Canavieiras (BA): análise dos conflitos inerentes à reprodução social e política das suas comunidades tradicionais. Desenvolvimento e Meio Ambiente 48:367-391. http://doi:10.5380/dma.v48i0.58675.

Duque-Rico, M.A., and L. Torres-Gómez. 2011. Aspectos socioeconómicos de las principales comunidades de los archipiélagos de San Bernardo y Nuestra Señora del Rosario, y del sector de Barú. In El entorno ambiental del Parque Nacional Natural Corales del Rosario y de San Bernardo, ed. E. Zarza-Gonzáles, 26-37. Cartagena de Indias: Quito Publicidad. (in Spanish)

Durán, C.A. 2009. Gobernanza en los Parques Nacionales Naturales colombianos: reflexiones a partir del caso de la comunidad Orika y su participación en la conservación del Parque Nacional Natural Corales del Rosario y San Bernardo. Revista de Estudios Sociales 32: 60-73.

Edgar, G.J., R.H. Bustamante, J.-M. Fariña, M. Calvopiña, C. Martínez, and M.V. Toral-Granda. 2004. Bias in evaluating the effects of marine protected areas: the importance of baseline data for the Galapagos Marine Reserve. Environmental Conservation 31(3): 212–218. https://doi:10.1017/S0376892904001584

Erler, D.M., D.P. Lima Junior, and A. Schiavetti. 2015. Ecological fishing networks in a marine protected area: One possibility for evaluating objectives. Ocean & Coastal Management 104: 106-114. https://doi:10.1016/j.ocecoaman.2014.12.008

Ernst, B., P. Manríquez, J.M. Orensanz, R. Roa, J. Chamorro, and C. Parada. 2010. Strengthening of a traditional territorial tenure system through protagonism in monitoring activities by lobster fishermen from Juan Fernández Islands (Chile). Bulletin of Marine Sciences 86: 315–338.

Esteves-Dias, A.C., A. Cinti, A.M. Parma, and C. Simão Seixas. 2020. Participatory monitoring of small-scale coastal fisheries in South America: use of fishers' knowledge and factors affecting participation. Reviews in Fish Biology and Fisheries 30: 313–333. https://doi:10.1007/s11160-020-09602-2

Ezcurra, E., O. Aburto-Oropeza, M. de los A. Carvajal, R. Cudney-Bueno, and J. Torre. 2009. Gulf of California, Mexico. In Ecosystem-based management for the oceans, eds. K.L. McLeod, and K. Leslie, 227–252. London: Island Press.

Fiorda, P., and A.M. Parma. 2015. Relevamiento de Bancos de Vieira Tehuelche en el Golfo San José. Centro Nacional Patagónico (CENPAT-CONICET), Technical Report 30, Puerto Madryn, Argentina. (in Spanish)

Fiorda, P., G. Trobbiani, and M. Parma. 2013. Relevamiento de Bancos de Vieira Tehuelche en el Golfo San José. Centro Nacional Patagónico (CENPAT-CONICET), Technical Report 29, Puerto Madryn, Argentina. (in Spanish)

Fulton S., J. Caamal-Madrigal, A. Aguilar-Perera, L. Bourillón, and W.D. Heyman. 2018. Marine conservation outcomes are more likely when fishers participate as citizen scientists: case studies from the Mexican Mesoamerican Reef. Citizen Science: Theory and Practice 3(1): 1–12. https://doi:10.5334/cstp.118

Gerber, L.R., J. Wielgus, and E. Sala. 2007. A decision framework for the adaptive management of an exploited species with implications for marine reserves. Conservation Biology 21(6): 1594–1602. https://doi:10.1111/j.1523-1739.2007.00824.x

Heylings, P., R. Bensted-Smith, and M. Altamirano. 2002. Zonificación e historia de la Reserva Marina de Galápagos. In: Reserva Marina de Galápagos, Línea Base de la Biodiversidad, eds E. Danulat, and G.J. Edgar, 10–21. Quito, Ecuador: Fundación Charles Darwin/Servicio Parque Nacional Galápagos.

Heylings, P., and M. Bravo. 2007. Evaluating governance: a process for understanding how co-management is functioning, and why, in the Galapagos Marine Reserve. Ocean & Coastal Management 50: 174–208. https://doi:10.1016/j.ocecoaman.2006.09.003

Heylings, P., and F. Cruz. 1998. Common Property, Conflict and Participatory Management in the Galapagos Islands. Conference Paper, 7th Biennial Conference of the IASC, Vancouver, Canada. Charles Darwin Research Station, Puerto Ayora, Galápagos, Ecuador.

Hockings, M., S. Valenzuela, M. Calvopiña, S. Chamorro, P. León, S. Bucaram, and M. Villalta. 2012. Galapagos Marine Reserve management effectiveness assessment. Galapagos: Dirección Parque Nacional Galápagos/World Wildlife Fund.

Incoder-UJTL. 2014. Visión integral de los Archipiélagos de Nuestra Señora del Rosario y de San Bernardo. Parte II. In Ambiente y Desarrollo en el Caribe colombiano. Publicación de la Universidad Jorge Tadeo Lozano Seccional Caribe 3 (2). (in Spanish)

INVEMAR-MADS. 2012. Plan de Manejo del Área Marina Protegida de los Archipiélagos de Rosario y San Bernardo AMP-ARSB (2013-2023). Santa Marta, Colombia: INVEMAR-MADS.

Ley-Cooper, K., S. De Lestang, B.F. Phillips, and E. Lozano-Álvarez. 2014. An unfished area enhances a spiny lobster, Panulirus argus, fishery: implications for management and conservation within a Biosphere Reserve in the Mexican Caribbean. Fisheries management and ecology 21(4): 264-274. https://doi:10.1111/fme.12072.

Lobão, R. 2010. Cosmologias Políticas do Neocolonialismo. Como uma política pública pode se transformar em uma política do ressentimento. Niterói: Editora da Universidade Federal Fluminense. (in Portuguese)

Loto, L. 2012. Reservas Extrativistas Marinhas vs. Áreas de Manejo e Exploração de Recursos Bentônicos: comparação de modelos de gestão compartilhada de áreas marinhas protegidas no Brasil e no Chile. Masters Thesis. Niterói: Universidade Federal Fluminense.

Llerena, E., T. Quisingo, and R. Maldonado. 2015. Análisis de los acuerdos logrados en la Junta de Manejo Participativo 2010-2015. In Informe Galápagos 2015-2016, eds. DPNG, CGREG, FCD and GC, 105-111. (in Spanish)

Maranhão, T.C. 2012. Significado da Pactuacão entre Órgãos Públicos Ambientais e Populações Tradicionais. Masters Thesis. Niterói: Universidade Federal Fluminense.

Marco, J., D. Valderrama, and M. Rueda. 2021. Evaluating management reforms in a Colombian shrimp fishery using fishery performance indicators. Marine Policy 125: 104258. https://doi:10.1016/j.marpol.2020.104258

Méndez-Medina, C., B. Schmook, and S.R. Mccandless. 2015. The Punta Allen cooperative as an emblematic example of a sustainable small-scale fishery in the Mexican Caribbean. Maritime Studies 14: 12. https://doi:10.1186/s40152-015-0026-9

Méndez-Medina, C., B. Schmook, X. Basurto, S. Fulton, and A. Espinoza-Tenorio. 2020. Achieving coordination of decentralized fisheries governance through collaborative arrangements: A case study of the Sian Ka'an Biosphere Reserve in Mexico. Marine Policy 117: 103939. https://doi:10.1016/j.marpol.2020.103939

Ministerio del Medio Ambiente y Unidad Administrativa Especial de Parques Nacionales Naturales (MMA-UAESPNN). 2001. Política de Consolidación del Sistema de Parques Nacionales Naturales 'Participación Social para la Conservación'. Bogotá, Colombia: MMA-UAESPNN. (in Spanish)

Ministerio de Ambiente y Desarrollo Sostenible (MADS). 2020. Plan de Manejo del Parque Nacional Natural Los Corales del Rosario y de San Bernardo. Resolución 0160. (in Spanish)

Ministerio de Ambiente y Desarrollo Sostenible (MADS). 2022. Plan de Manejo del Área Marina Protegida de los Archipiélagos del Rosario y de San Bernardo. Resolución 0551. (in Spanish)

Neira, A., J.M. Díaz., G. González, M.C. Velandia, and G. Melo. 2016. Diagnóstico de las pesquerías artesanales en el norte del Pacífico chocoano. In La pesca artesanal en la costa norte del Pacífico colombiano: un horizonte ambivalente, eds. J.M. Díaz, L. Guillot and M.C. Velandia, 69-89. Bogotá: Fundación MarViva.

Orensanz, J.M., A. Cinti, A.M. Parma, L. Burotto, S. Espinosa-Guerrero, E. Sosa-Cordero, C. Sepulveda, and V. Toral-Granda. 2013. Latin-American rights-based fisheries targeting sedentary resources. In Rights-based management in Latin American fisheries, eds. J. Orensanz, and J. Seijo, 72–134. FAO Fisheries and Aquaculture Technical Paper 582, Rome, Italy.

Orensanz, J.M., A.M. Parma, and A. Cinti. 2015. Methods to use fishermen' knowledge for fisheries assessment and management. In Fishermen' knowledge and the ecosystem approach to fisheries: applications, experiences and lessons in Latin America, eds. J. Fischer, J. Jorgensen, H. Josupeit, D. Kalikoski, and C.M. Lucas, 41-62. FAO Fisheries and Aquaculture Technical Paper 591. Rome: FAO.

Orensanz, J.M., A.M. Parma, N.F. Ciocco, and A. Cinti. 2003. Programa de entrada limitada para la pesca comercial de mariscos mediante buceo en el Golfo San José. Technical Advisory Board for the Management of the Commercial Diving Fishery, Chubut Province, Technical Report 9, Puerto Madryn, Argentina. (in Spanish)

Orensanz, J.M., A.M. Parma, N.F. Ciocco, and A. Cinti. 2007. Commercial diving for bivalves in San José Gulf, Argentine Patagonia- Round 5 is on. In Fisheries Management: Progress Towards Sustainability, eds. T.R. McClanahan, and J.C. Castilla, 68–87. Oxford: Blackwell Publishing.

Orensanz, J.M., A.M. Parma, G. Jerez, N. Barahona, M. Montecinos, and I. Elías. 2005. What are the key elements for the sustainability of "S-fisheries"? Insights from South America. Bulletin of Marine Science 76: 527–556.

Parma, A.M., J.M. Orensanz, I. Elías, and G. Jerez. 2003. Diving for shellfish- and data: incentives for the participation of fishers in the monitoring and management of artisanal fisheries around southern South America. In Towards sustainability of data-limited multi-sector fisheries, eds. S.J. Newman, D.J. Gaughan, G. Jackson, M.C. Mackie, B. Molony, J. St John, and P. Kaiola, 8-29. Australian Society for Fish Biology Workshop Proceedings, Bunbury, Australia.

Pellowe, K.E., and H.M. Leslie. 2020. The interplay between formal and informal institutions and the potential for co-management in a Mexican small-scale fishery. Marine Policy 121: 104179. https://doi:10.1016/j.marpol.2020.104179

Penchel-Araújo, V.P., and O. Souza-Nicolau. 2018. Participação social na Reserva Extrativista Marinha de Arraial do Cabo: uma análise dos instrumentos de gestão sob a ótica da descolonialidade. Desenvolvimento e Meio Ambiente 48: 299-320. https://doi:10.5380/dma.v48i0.58831.

Peterson, N.D. 2011. Excluding to include: (Non)participation in Mexican natural resource management. Agriculture and Human Values 28: 99–107. https://doi:10.1007/s10460-010-9258-x

Ramírez, L. 2016. Marine Protected Areas in Colombia: Advances in Conservation and Barriers for Effective Governance. Ocean & Coastal Management 125: 49-62. https://doi:10.1016/j.ocecoaman.2016.03.005

Ramírez, L. 2017. Marine Protected Areas in Colombia: Re-connecting Social, ecological, and policy aspects through a governance perspective. PhD Thesis. Waterloo, Canada: Wilfrid Laurier University.

Ramírez-Sánchez, S., and E. Pinkerton. 2009. The Impact of Resource Scarcity on Bonding and Bridging Social Capital: the Case of Fishers' Information-Sharing Networks in Loreto, BCS, Mexico. Ecology and Society 14(1): 22. https://www.ecologyandsociety.org/vol14/iss1/art22/

Rife, A.N., B. Erisman, A. Sánchez, and O. Aburto-Oropeza. 2013. When good intentions are not enough … Insights on networks of "paper park" marine protected areas. Conservation Letters 6 (3): 200-212. https://doi:10.1111/j.1755-263X.2012.00303.x

Rodríguez, A., M. Rueda, T. Viaña, C. García, F. Rico, and L. García. 2012. Evaluación y manejo de la pesquería de camarón de aguas profundas en el Pacífico colombiano 2010-2012. Serie de Documentos Generales INVEMAR 56. Santa Marta, Colombia: INVEMAR, COLCIENCIAS, INCODER. http://hdl.handle.net/1834/6661

Roldán, A.M. 2013. Alternativas y retos para la gobernanza de la pesca artesanal: una revisión al enfoque de manejo basado en derechos para el Pacífico colombiano. Masters Thesis. Bogotá, Colombia: Pontificia Universidad Javeriana. (in Spanish)

Rueda, M., O. Doncel, E.A. Viloria, D. Mármol, C. García, A. Girón, L. García, F. Rico, et al. 2011. Atlas de la pesca marino-costera de Colombia: 2010-2011. Tomo Caribe. INVEMAR y ANH. Serie de publicaciones del INVEMAR. (in Spanish)

Rueda, M., J. Viaña, S. Salas, A. Girón, D. Rubio-Lancheros, D. Bustos-Montes, and F. Escobar-Toledo. 2020. Construcción participativa de acuerdos de pesca sostenible en la pesquería de

arrastre de camarón en el Pacífico de Colombia. Serie de publicaciones generales del Invemar 114. (in Spanish)
Saavedra-Díaz, L.M., A.A. Rosenberg, and B. Martín-López. 2015. Social perceptions of Colombian small-scale marine fisheries conflicts: Insights for management. Marine Policy 56: 61–70. https://doi:10.1016/j.marpol.2014.11.026
Seijo, J.C. 1993. Individual transferable grounds in a community managed artisanal fishery. Marine Resource Economics 8(1): 78–81. https://doi:10.1086/mre.8.1.42629048.
Soria, G., P. Fiorda, A.M. Parma, and L.M. Getino-Mamet. 2017. Relevamiento de bancos de vieira Tehuelche en el Golfo San José-SANJO2017. Centro Nacional Patagónico (CENPAT-CONICET), Technical Report, Puerto Madryn, Chubut. (in Spanish)
Sosa-Cordero, E., and A. Ramírez-González. 1993. El uso de hábitats artificiales en la pesquería de langosta Panulirus argus de Quintana Roo, México. In Memorias del I Taller Bilateral México-Cuba, eds. J. González-Cano, and R. Cruz, 142-149. Mexico City: SEPESCA-INP. (in Spanish)
Sosa-Cordero, E., M.L.A. Liceaga-Correa, and J.C. Seijo. 2008. The Punta Allen lobster fishery: current status and recent trends. In Case studies in fisheries self-governance, eds. R. Townsend, R. Shotton, and H.C. Uchida, 149–162. Rome: FAO.
Stamieszkin, K., J. Wielgus, and L.R Gerber. 2009. Management of a marine protected area for sustainability and conflict resolution: Lessons from Loreto Bay National Park (Baja California Sur, Mexico). Ocean & Coastal Management 52(9): 449-458. https://doi:10.1016/j.ocecoaman.2009.07.006
Steinitz, C., R. Faris, J.C. Vargas-Moreno, G. Huang, S-Y Lu, O. Arizpe, M. Angeles, F. Santiago, et al. 2005. Alternative futures for the region of Loreto, Baja California Sur, Mexico. Harvard University, Report, Cambridge, USA.
Subsecretaría de Pesca y Acuicultura (SUBPESCA). 2014. Bases para la creación de Red de Parques Marinos Archipiélago Juan Fernández. Technical Report 19, Valparaíso, Chile. (in Spanish)
Taver-Escobar, H.A, H. Sánchez-Páez, G.A. Ulloa-Delgado, and A. Zamora-Guzmán. 2004. Plan de manejo integral de los manglares de la Zona de Uso Sostenible de la ciénaga de La Caimanera, Sucre – Colombia. Sincelejo, Colombia: MAVDT/CONIF/CARSUCRE. (in Spanish)
Villasante, S., I. Gianelli, M. Castrejón, L. Nahuelhual, L. Ortega, U. Rashid Sumaila, and O. Defeo. 2022. Social-ecological shifts, traps and collapses in small-scale fisheries: Envisioning a way forward to transformative changes. Marine Policy 136: 104933. https://doi:10.1016/j.marpol.2021.104933
Wielgus, J., F. Ballantyne, E. Sala, and L.R. Gerber. 2007. Viability analysis of reef fish populations based on limited demographic information. Conservation Biology 21(2): 447–454. https://doi:10.1111/j.1523-1739.2006.00644.x
Wielgus, J., E. Sala, and L.R. Gerber. 2008. Assessing the ecological and economic benefits of a no-take marine reserve. Ecological Economics 67(1): 32-40. https://doi:10.1016/j.ecolecon.2008.04.019
